# Collective Genetic Interaction Effects and the Role of Antigen-Presenting Cells in Autoimmune Diseases

**DOI:** 10.1371/journal.pone.0169918

**Published:** 2017-01-12

**Authors:** Hyung Jun Woo, Chenggang Yu, Jaques Reifman

**Affiliations:** Biotechnology High-Performance Computing Software Applications Institute, Telemedicine and Advanced Technology Research Center, U.S. Army Medical Research and Materiel Command, Maryland, United States of America; Johns Hopkins University, UNITED STATES

## Abstract

Autoimmune diseases occur when immune cells fail to develop or lose their tolerance toward self and destroy body’s own tissues. Both insufficient negative selection of self-reactive T cells and impaired development of regulatory T cells preventing effector cell activation are believed to contribute to autoimmunity. Genetic predispositions center around the major histocompatibility complex (MHC) class II loci involved in antigen presentation, the key determinant of CD4^+^ T cell activation. Recent studies suggested that variants in the MHC region also exhibit significant non-additive interaction effects. However, collective interactions involving large numbers of single nucleotide polymorphisms (SNPs) contributing to such effects are yet to be characterized. In addition, relatively little is known about the cell-type-specificity of such interactions in the context of cellular pathways. Here, we analyzed type 1 diabetes (T1D) and rheumatoid arthritis (RA) genome-wide association data sets via large-scale, high-performance computations and inferred collective interaction effects involving MHC SNPs using the discrete discriminant analysis. Despite considerable differences in the details of SNP interactions in T1D and RA data, the enrichment pattern of interacting pairs in reference epigenomes was remarkably similar: statistically significant interactions were epigenetically active in cell-type combinations connecting B cells to T cells and intestinal epithelial cells, with both helper and regulatory T cells showing strong disease-associated interactions with B cells. Our results provide direct genetic evidence pointing to the important roles B cells play as antigen-presenting cells toward CD4^+^ T cells in the context of central and peripheral tolerance. In addition, they are consistent with recent experimental studies suggesting that the repertoire of B cell-specific self-antigens in the thymus are critical to the effective control of corresponding autoimmune activation in peripheral tissues.

## Introduction

Autoimmune diseases [1], such as type 1 diabetes (T1D) [2], rheumatoid arthritis (RA) [3], and multiple sclerosis [4], arise from the inadequate control of immune cell reactivity toward self-antigens and the resulting destruction of target organs. In both T1D and RA, genome-wide association studies (GWAS) have revealed dominant effects of the major histocompatibility complex (MHC) region, whose polymorphisms affect MHC class II antigen presentation and recognition [5–7]. Many additional loci, discovered from studies using genome-wide array and Immunochip designs [8–10], reinforce this picture by revealing the disease association of numerous receptors and regulators mediating such interactions, including, for instance, *PTPN22* and *CTLA4* [5, 7].

In T1D, the autoimmune action takes the form of T cells infiltrating the pancreas and destroying insulin-producing β-cells. Although the presence of autoantibodies indicates that humoral immunity contributes to this late-stage pathogenesis [2, 11, 12], this mechanism also depends on activation by cognate CD4^+^ T cells. RA, characterized by inflammations affecting small joints of hands and feet, occurs when T cells, B cells, and macrophages enter the synovium and destroy local tissues [3]. Evidence suggests that the B cell receptor (BCR)-mediated antigen presentations by B cells in the periphery are critical for the activation of these cognate CD4^+^ T cells in both T1D [13] and RA [14, 15]. Important roles B cells play have also been established in other autoimmune diseases including systemic lupus erythematosus [16].

The helper T cells (T_h_) specific to self-antigens originate from the thymus, where the immature T cell repertoires are first selected for moderate self-reactivity (positive selection) by cortical thymic epithelial cells (cTECs) [17]. The subsequent negative selection of these cells in the medulla depends on the strength of interactions with a range of antigen-presenting cells (APCs) [18], which include medullary thymic epithelial cells (mTECs) and dendritic cells (DCs). The mTECs promiscuously express tissue-restricted antigens (TRAs), including insulin, promoted by the transcription factor AIRE. These antigens are either presented by mTECs themselves or “handed-over” to DCs for presentation on MHC class II molecules toward immature T cells. Strongly reactive T cell subsets are subsequently led to apoptosis. Recent studies suggested that in addition to mTECs and DCs, thymic B cells can also act as APCs [19], expressing AIRE and TRAs [20]. B cells therefore appear to act as APCs both in thymic selection and in the peripheral activation of T_h_ cells, which presumably reflect the need to train T cell populations in the thymus against the antigen repertoire specific to B cell presentation in the periphery [20].

This clonal deletion, however, is incomplete, and many T cells migrating into peripheral tissues are now known to be self-reactive even in healthy individuals [21]. The deleterious effects of auto-reactivity are kept in check by the suppressive action of regulatory T cells (T_reg_), whose natural variant originates from differentiation of immature T cells in the thymus [22]. These T_reg_ cells migrate into peripheral lymphoid organs and suppress the activation of self-reactive effector cells [23]. The current consensus is that both negative selection (the likely fate of T cells with stronger affinity to self-antigens) and T_reg_ cell induction (more likely for those with intermediate affinity range) in the thymus during the prenatal time period are crucial for the effective control of auto-reactivity in peripheral tissues [21].

Tracking down the exact cellular and molecular events in these two aspects of tolerance (negative selection and T_reg_ differentiation) is key to the development of intervention and treatment strategies against autoimmune diseases [11]. It is currently not clear, for instance, to what extent different cell types with the capacity to act as APCs (mTECs, DCs, thymic and peripheral B cells) individually contribute to these processes. We show in this work that in addition to cell biological methods using transgenic mice, analyses of genetic data can also shed light on such issues. Our approach is based on enhanced inference of epistatic effects between genetic factors, which likely play important roles in heritability components undetectable from single-variant analyses [24]. Many recent studies pointed to the importance of non-additive interaction effects in autoimmune disease associations within the dominant MHC loci and among the classical HLA alleles [25, 26]. Large-scale collective interactions involving multiple MHC SNPs, however, remain to be characterized. Furthermore, it is of interest to identify the types of lymphocytes or tissues in which these interactions occur, taking into account tissue-specific epigenetic modifications of the genetic loci: many disease-associated single-nucleotide polymorphisms (SNPs) are in the non-coding region, presumably exerting their effects via changes to gene regulatory mechanisms, which are often cell-type-specific [9].

In this work, we tested the following hypotheses relevant to the cell-type-specific genetic effects of autoimmune risks discussed above:

1. Non-additive, collective interaction effects associated with disease status reflect cellular interaction mechanisms underlying pathogenesis.
2. Interrogation of the spatially resolved interaction patterns allows for the identification of specific genetic loci responsible for such interactions.

The first hypothesis is an extension of its counterpart implicit in standard non-interacting SNP analyses: disease-associated loci identified by independent-SNP treatments reveal the effects of genes or non-coding regulatory factors participating in pathogenesis mechanisms, which can be inferred from their cell-type-specificity. Analyses incorporating explicit interaction effects can reveal higher-order effects, such as interaction of two genetic factors, each expressed highly in different cell types (e.g., T cells and their APCs). The second hypothesis is an extension of the fine-mapping or conditional analysis targeting a causal SNP or spatial region within a loci masked by linkage disequilibrium (LD).

We addressed these questions by using a recently developed algorithm, discrete discriminant analysis (DDA) [27], to infer non-additive interaction effects involving a large number of SNPs in association with autoimmune disease risks. The results revealed that in addition to exhibiting extensive LD, the MHC region also possesses *differential* LD patterns between case and control groups, which translate into collective disease associations caused by a large number of SNPs acting cooperatively. We interpreted such collective effects on disease risks in cell-type/tissue-specific manner, addressing the question of how different APCs interact with thymic and peripheral CD4^+^ T cells and how disease-associated genes interact within the context of T cell signaling and differentiation. We analyzed two representative autoimmune disease data sets (T1D and RA) from the Wellcome Trust Case-Control Consortium (WTCCC) study [6] and assessed the outcomes by comparison with the summary statistics of more recent meta-analysis data [8, 10]. The use of relatively smaller but well-characterized data sets facilitated computational analysis necessary for DDA, which optimizes all possible simultaneous interactions involving many SNPs while testing for significance of interacting pairs. We first focused on the MHC region, where instead of attempting to pinpoint a few SNP pairs with high significance, we extracted a large pool of SNP pairs with moderate degrees of association and examined the enrichment of epigenetically active subsets. We found robust epigenome-specific interaction patterns common to both T1D and RA, which suggested that B cells acting as APCs are the main sources of genetic predispositions reflected in MHC loci. This observation supports experimental evidence for the role of B cells as APCs in general [20] and in RA as shown by Taneja and co-workers [15]. We further complemented this analysis by selecting SNPs genome-wide based on biological pathways for DDA collective inference analysis.

## Results

### Independent-SNP analysis

**Fig 1:**
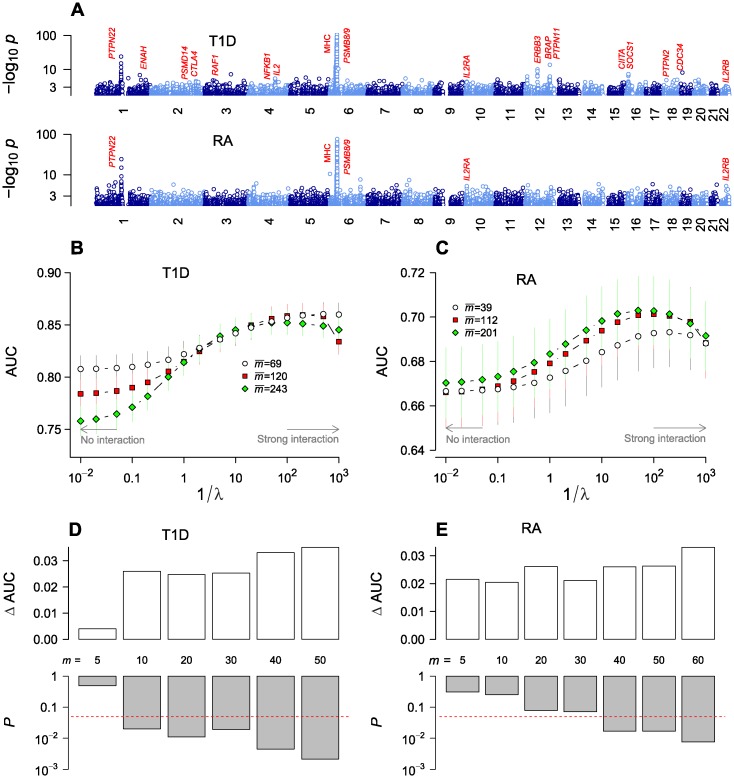
Genome-wide association of type 1 diabetes (T1D) and rheumatoid arthritis (RA) single-nucleotide polymorphisms (SNPs) under independent-SNP and collective inferences. (**A**) Independent-SNP *p*-value profiles based on genotypic model. Note that the *p*-values are in “double logarithmic” scale for clarity. (**B-C**) Optimization of collective inference model parameters with cross-validation area under the curve (AUC) as a function of model sizes (mean number of SNPs selected) and inverse of penalizer 1/λ. The small and large-1/λ limits correspond to non-interacting and strongly interacting limits, respectively. Vertical bars are 95% c.i. (**D-E**) Statistical significance of the rise in AUC from interaction effects (ΔAUC; defined as the difference between the maximum AUC and the non-interacting limit). For T1D (**D**) and RA (**E**), the first *m* SNPs were selected from the sorted list with increasing order of single-SNP *p*-value *p*_*i*_, and the null distribution of ΔAUC was sampled by permutation of the phenotypic label to estimate the *p*-value (*bottom*; horizontal line represents *p* = 0.05) for the significance of the actual ΔAUC observed (*top*).

We first analyzed the WTCCC T1D genome-wide data using the independent-SNP special case of the DDA algorithm. This special case with interactions turned off yields results closely matching logistic regression-based analyses [27] and allows for the identification of an initial candidate set of statistically significant SNPs. The genome-wide scan was consistent with the original report [6] (Fig 1A), dominated by the MHC region and, for T1D, with additional signals near *PTPN22* on chromosome 1 and loci on chromosomes 12 and 16. *CTLA4* (strongest SNP rs231726, independent-SNP *p*-value *p*_*i*_ = 5×10^−6^), *IL2RA* (rs10795791, *p*_*i*_ = 4×10^−5^), and *INS* loci (rs6578252, *p*_*i*_ = 2×10^−4^) had *p*-values below genome-wide significance. The *p*_*i*_ profile for RA was similar to T1D but with significantly weaker association strengths: the bulk of genetic factors was also explained by the MHC group, with the second robustly identified locus near *PTPN22*.

### Cross-validation

Using the quality-controlled whole-genome SNP set, we performed cross-validation to assess the inference performance with varying numbers of selected SNPs and determined the optimal degree of interactions to be included by varying the parameter λ. The overall quality of model fit is represented by the area under the curve (AUC) of the receiver operating characteristics, a measure ranging from 1.0 to ~0.5, with decreasing sensitivity and specificity. We selected SNPs (*m* in total number in each run) with *p*_*i*_ below a cutoff. The penalizer λ values of 0 and ∞ correspond to the limits of strongest interactions and no interaction, respectively. In Fig 1B and 1C, AUC is plotted as a function of 1/λ such that the non-interacting limit is reached on the left and interaction strength increases with increasing 1/λ.

For T1D, we found high AUC values (~0.85) in the strongly interacting regime (large 1/λ), consistent with the general observation that T1D association is one of the strongest among common diseases [5] (Fig 1B). The non-interacting limit showed an AUC range of 0.75~0.80, which is similar to values reported previously in the literature for predictive models (polygenic risk scoring) without interaction [28]. The maximum AUC values were lower than but comparable to those reported (~0.89) using support vector machines [29]. We also varied model sizes (number of SNPs included) and observed lower AUCs when the mean SNP number increased beyond ~100, which implies that the model performance is dominated by SNPs with the strongest association (MHC region; Fig 1A) and that including extra variants purely based on *p*_*i*_ led to a net dilution of prediction performance. Analogous cross-validation for RA data (Fig 1C) showed clear maxima near 1/λ ~ 100, with the maximal values rising moderately from 0.69 (*p*_*i*_ < 10^−20^; mean SNP number 39) to 0.70 (*p*_*i*_ < 10^−5^; mean SNP number 201) with increasing model sizes. The absolute AUC values were lower than in T1D, reflecting a weaker overall strength of association under similar sample sizes.

We evaluated the statistical significance of the maximum AUC values relative to the non-interacting limit by considering it as a statistic and sampling its null distribution with phenotype label permutation (Fig 1D and 1E). The SNP number *m* was varied by selecting those with the lowest *p*_*i*_. The AUC change due to interaction (ΔAUC) rose, on average, with increasing *m*. The corresponding *p*-values decreased with increasing *m*, becoming smaller than the nominal *p* = 0.05 for *m* ≥ 10 (T1D) and *m* ≥ 40 (RA).

### Collective interaction analysis

**Fig 2:**
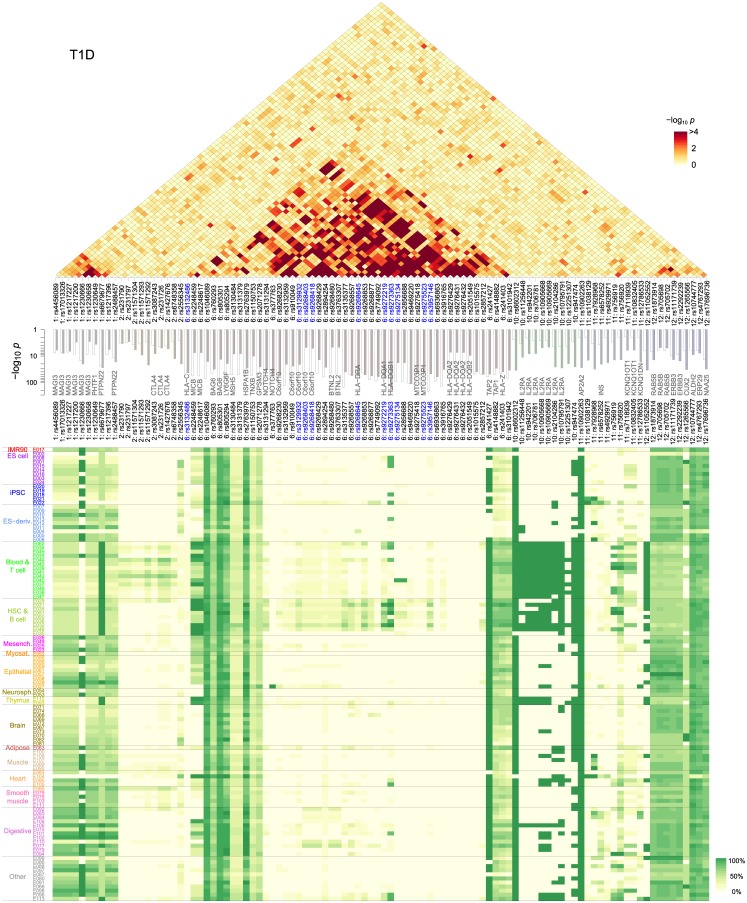
T1D collective inference *p*-values and epigenetic activity distribution. SNPs (“proxies”) were selected based on their independent-SNP *p*_i_ values (*middle*, open bars). The *top* (triangular) and *middle* (bar graph) panels show the interaction and (additive) single-SNP contributions, respectively. Bottom panel shows the mean frequencies (averaged over groups of all known SNPs in high LD to each proxy) of epigenetically active states within the 111 Roadmap reference epigenomes.

We then aimed to derive subsets of SNPs potentially exerting significant interaction effects for further analysis based on independent-SNP *p*-value profiles. For T1D, selecting SNPs using *p*_*i*_ < 10^−30^ yielded *m* ~ 100 SNPs only from the MHC region. We sought to include possible interactions with non-MHC loci by selecting 50 SNPs from the MHC region and 10 each from *PTPN22*, *CTLA4*, *INS*, *IL2RA*, and 12q13/12q24 (“chr-12”) loci [6], respectively. We compiled the lists of known SNPs [30] in strong LD (*r*^2^ > 0.5) with these “proxy” SNPs, which covered *PTPN22*, *CTLA4*, MHC class III and II regions, and a part of *IL2RA* gene (S1 Fig); the SNP group closest to the *INS* gene (proxy: rs6578252) was ~40 kb downstream from it on chromosome 11p15.5, partially explaining the lack of *INS* association from the WTCCC data. We then derived collective inference parameters for this SNP selection using the optimal penalizer value (λ = 0.01) suggested by cross-validation (Fig 1B) and tested the significance of single-SNP and interaction parameters (Fig 2).

The single-SNP *p*-values shown in Fig 2 (*middle*) represent the significance of the *additive* part of the model association. These values reduce to the independent-SNP *p*_*i*_ (Fig 2, *middle*, open bars, and Fig 1A) when interactions are turned off [27]. When inferred with interactions, these single-SNP *p*-values in the MHC group were significantly reduced (Fig 2, *middle*, solid bars), while many of the non-MHC SNPs were enhanced. The SNP with the smallest single-SNP *p*-value (rs9273363, *p* ~ 10^−174^) remained the same with interaction as in the independent-SNP case. This SNP is ~1 kb upstream from *HLA-DQB1* gene and in LD with rs3830060 (*r*^2^ = 0.517) located in the coding region. The observation of highest T1D association with SNPs near the *HLA-DQB1* gene is in agreement with fine-mapping studies [26]. The shift of association signals into interactions was drastic for some SNPs: two of them (rs9268480 and rs3763307) near *BTNL2* gene showed *p*_*i*_ ~ 10^−45^, which shifted to *p* ~ 10^−5^ with interaction. Another example is provided by four SNPs (rs9276429, rs9276431, rs9276432, and rs2051549) in *HLA-DQA2* and *HLA-DQB2* that showed shifts from *p* < 10^−44^ (non-interacting) to *p* ~ 10^−8^ (collective). The effects of these SNPs are expected to arise primarily from interactions. In contrast, many variants outside MHC showed enhanced single-SNP *p*-values.

We also noted relatively strong associations from four SNPs (rs241427, rs4148882, rs241403, and rs3101942) near *TAP1/TAP2* and *PSMB8/PSMB9*. *TAP1/2* encode protein subunits transporting antigen peptides into endoplasmic reticulum (ER) in the MHC class I antigen presentation pathway [31], whereas *PSMB8/9* code for immune cell-specific versions of the catalytic subunits (β5i and β1i) of proteasomes that degrade peptides. The proxy SNP rs241427 (and three SNPs in LD) resided inside *TAP2*, whereas rs4148882, rs241403, rs3101942 all had similar distributions of SNPs in LD that extended from the 3’-end of *TAP2* to the region close to *HLA-DMB* encompassing *PSMB8/9* and *TAP1* (S2 Fig).

The changes in the single-SNP *p*-values were offset by extensive interaction patterns, notably within the MHC region, reflected in the interaction *p*-value landscape (Fig 2, *top*). One of the key sub-regions was near *HLA-DQA1* and *HLA-DQB1* (rs9272219 and rs9273363), whose protein products form the MHC class II heterodimer on the surface of APCs that display antigens toward the T cell receptor (TCR). *HLA-DQB1* encodes the DQ β-chain that interacts directly with TCR, and we deduce that a significant part of its dominant role in T1D association stems from interactions. We compared these interaction patterns with LD (S3 Fig). Interactions between SNPs inferred from DDA manifest themselves as *differences* in allele-frequency correlations between case and control groups: if LD patterns within case and control groups were identical, the difference would vanish even when individual correlations are large. For instance, although there was a strong LD within the *CTLA4* and *IL2RA* loci in S3 Fig, the corresponding interactions were largely absent (Fig 2).

**Fig 3:**
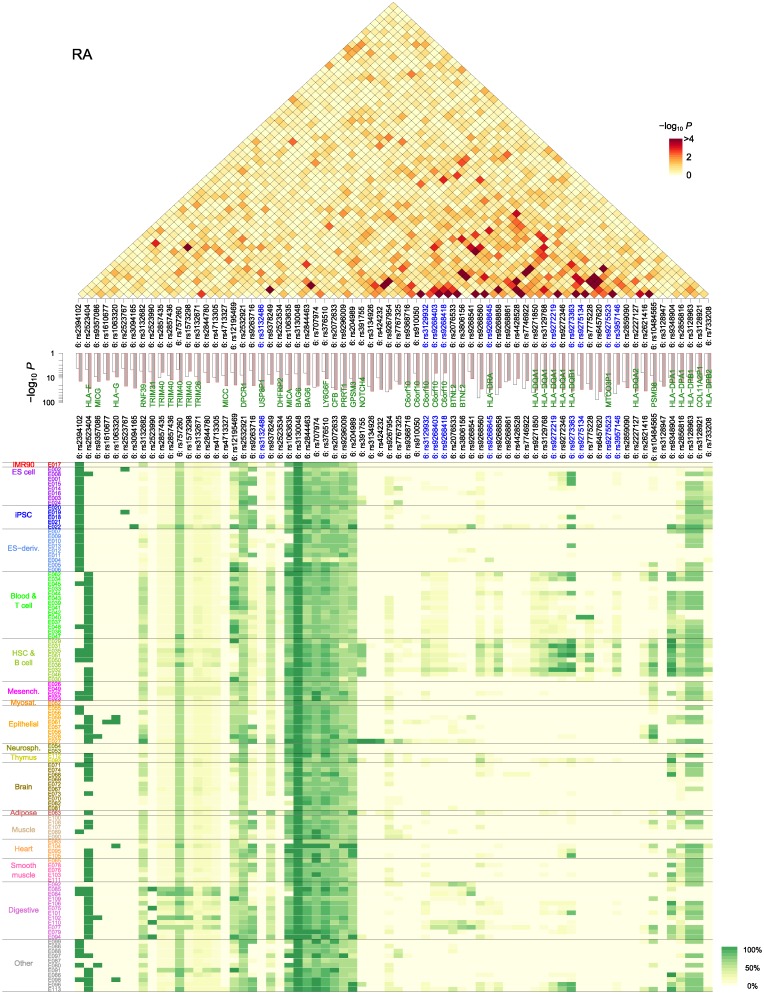
RA collective inference *p*-values and epigenetic activity distribution. *m* = 70 proxy SNPs were selected based on *p*_i_ values (*middle*, open bars) combined with clustering to reduce LD. The *top* (triangular) and *middle* (bar graph) panels show the interaction and (additive) single-SNP contributions, respectively. Bottom panel shows the mean frequencies of epigenetically active states as in Fig 2.

For RA, we used a different SNP selection method to examine whether strong LD in the MHC region affected the outcome: we first selected a larger number (*m* = 255) of SNPs and used clustering to filter them into a selection of *m* = 70 SNPs. Compared to T1D, this RA selection had significantly reduced redundancy of SNPs with similar LD patterns, and included ~20 SNPs in the MHC class I region (Fig 3). We inferred additive single-SNP and interaction *p*-values of the selected model: in the non-interacting case (Fig 3, *middle*, open bars), strongest association was observed within the selection at rs6457620 (*HLA-DQB1*; *p*_*i*_ = 3×10^−77^), rs9268560 (*HLA-DRA*; *p*_*i*_ = 5×10^−66^), and rs2076533 (*BTNL2*; *p*_*i*_ = 3×10^−60^). In comparison, when interactions were included, single-SNP *p*-values were more uniformly distributed (Fig 3, *middle*, solid bars). SNP pairs with low interaction *p*-values were less concentrated in the MHC class II region than in T1D (Fig 2), reflecting the smaller number of SNPs in high LD.

### Cell-type-specific effects of variants

The results described above allow us to test our first hypothesis regarding the cell-type-specificity of disease-associated SNP interactions. For this purpose, we superimposed the single-SNP and interaction maps on the epigenetic-state annotations of the corresponding genomic locations in cellular contexts [27]. We used reference epigenomes from the National Institutes of Health Roadmap Epigenomics data [32], classifying their genomic annotations into *active* (transcribed or enhancer) and *inactive* states. These annotations, inferred from machine learning of chromatin modification and DNA accessibility data, should be regarded only as first approximations of the actual activity of genomic loci. We also accounted for the fact that our proxy SNPs likely reflect true causal SNPs indirectly via LD by averaging the active state frequencies over the list of all known SNPs in LD (S1 and S4 Figs). The resulting map (Fig 2, *bottom*; see S5 Fig for epigenome codes of each cell type [32]) suggested that a significant part of the MHC SNPs (class II region including *HLA-DR* and *HLA-DQ*) were highly specific, active largely in immune cells and digestive tissues (fetal stomach/intestine, colon, rectal/stomach/duodenum mucosa; S5 Fig) only. In contrast, the class III region, *PTPN22*, and chr-12 loci were uniformly active. The SNPs in *CTLA4* were active only in T cells, whereas *IL2RA* variants were active in both T and B cell groups, the latter including monocytes (progenitors of DCs) and natural killer cells (S5 Fig). The RA map (Fig 3, *bottom*) included SNPs near the *HLA-DP* genes, which were more broadly active than the other class II loci. Overall, the epigenetic activity distributions suggested that the dominant part of single-SNP contributions from the MHC class II region mostly involve the action of B and T cells and a subset of digestive mucosal cells.

**Fig 4:**
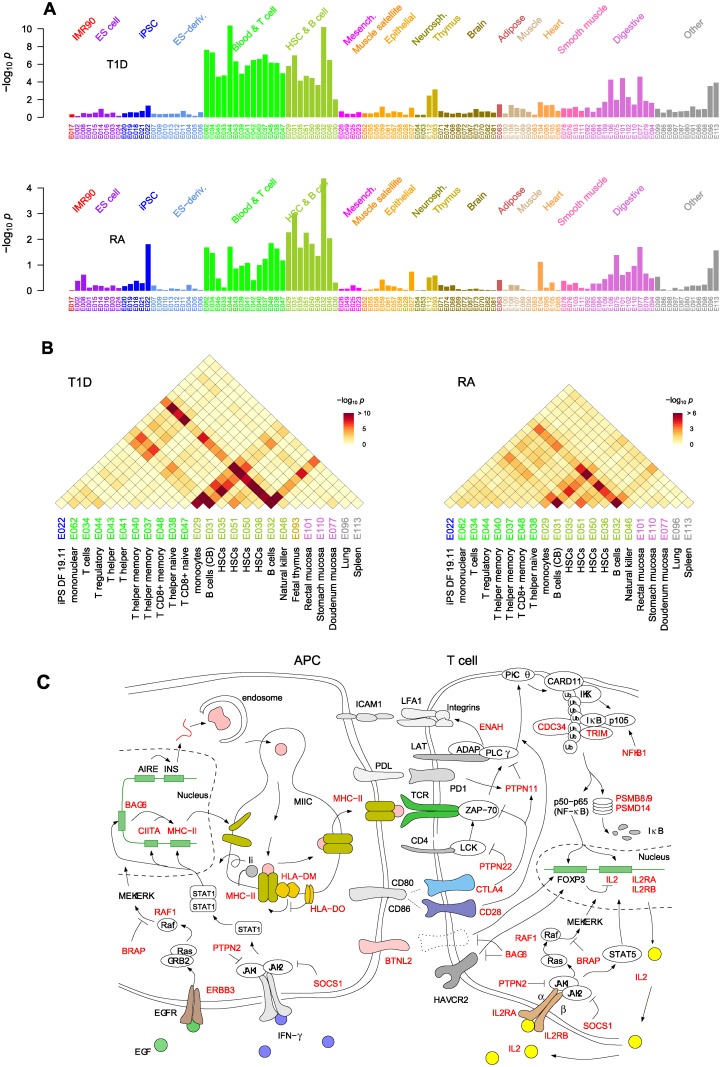
Cell type-specific enrichment of SNPs and their interactions. (**A**) Single-SNP enrichment (over-representation *p*-values) of T1D and RA-associated proxy SNPs (Fig 2) in reference epigenomes. (**B**) Enrichment of statistically significant interaction pairs active in cell type combinations. Combinations with negligible enrichment are not shown for clarity. Note the dominance of B cells in their interaction to T cells, monocytes, and intestinal epithelial cells. See S5 Fig for the full epigenome names. (**C**) Interrelationships of genetic factors associated with autoimmunity in the context of antigen presenting cell (APC) versus T cell interaction. Thymic APCs are stimulated by the epidermal growth factor receptor (EGFR) and interferon (IFN)-γ signaling pathways to express major histocompatibility (MHC) class II molecules, which are assembled in the endoplasmic reticulum (ER) and loaded by AIRE-induced self-peptides in the endosomal MHC class II compartment (MIIC). The peptide-MHC complex is recognized by the T cell receptor (TCR), initiating its downstream signaling leading to the activation of NF-κB, which enters the nucleus and up-regulates *FOXP3* (in T_regs_), interleukin (IL)-2 receptors, and IL-2 (in conventional CD4^+^ T cells). IL-2 signaling is crucial to both conventional T cell proliferation and T_reg_ cell activation. Genes shown in red are those implicated by the top-ranked pathways (Fig 6).

To infer quantitative measures of cell-type-specific association, we then performed enrichment analyses of active SNPs and their interactions. We first calculated the enrichment *p*-values for the over-representation of active SNPs among individual proxy SNP sets within different epigenomes (Fig 4A) and found strong enrichment in T cells and B cells: for T1D, the highest enrichment was with T_reg_ cells (E044) and B cells (E032) in these two groups. The overall enrichment was stronger with T1D than RA (geometric means of *p*-values over T and B cell groups were 1.1×10^−6^ and 0.031 for T1D and RA, respectively). This comparison exemplified a general trend we observed (from Figs 1–3): significantly higher effect sizes and power in T1D data than in RA, but broad qualitative similarities across the two diseases. The single-SNP enrichment distribution also included marginal signatures in the thymus, a number of digestive tissues (E106: sigmoid colon, E101: rectal mucosa, and E077: duodenum mucosa), in addition to the lung (E096) and spleen (E113).

We then sought to delineate how interacting SNP pairs (Figs 2 and 3) were represented in different cell type combinations. The enrichment *p*-value landscapes in Fig 4B signify the over-representation of active state-active state pairs among interacting SNP pairs in different cell type combinations. The most prominent were those with peripheral blood B cells (E032): SNPs active in B cells interacted with those active in a wide range of T cells, hematopoietic stem cells, and digestive tissues, in addition to the lung and spleen. The B cells from cord blood showed similar patterns with weaker effects. Despite considerable differences in SNP selection and the resulting interaction *p*-value distributions between T1D (Fig 2) and RA (Fig 3), their enrichment patterns (Fig 4B) were strikingly similar, suggesting a common underlying mechanism of autoimmune responses.

The repertoire of cell types with enriched interaction reflected the lineage of lymphocytes involved: hematopoietic stem cells (E035, E051, E050, and E036) differentiate into immune cells, including B cells (E032 and E031), T cells (E034) in the thymus (E093), and monocytes (E029), the progenitors to DCs. The fetal thymus (E093) featured weakly in the T1D landscape, interacting with B cells, whereas the adult thymus (E112) did not. This observation is consistent with the fact that the bulk of T cell maturation in the thymus occurs during the prenatal phase, and fetal and adult thymi secrete distinct lineages of T cells [33]. Among the three different classes of APCs, mTECs and DCs are expected to be reflected in the fetal thymus (E093) and monocytes (E029) epigenomes, respectively. We found that T cell interactions with monocytes and B cells were significantly enriched but the corresponding interaction with fetal thymus was not (Table 1). B cells from peripheral blood showed stronger enrichment than cord blood; Yamano et al. recently reported that peripheral B cells immigrate into the thymus and are transformed into thymic B cells by CD40 licensing signals from CD4^+^ T cells [20]. T cells that strongly interacted with DCs and B cells comprised naive T_h_ cells (E038) and T_reg_ cells, which is consistent with the view that both clonal deletion and T_reg_ induction in the thymus are crucial for the control of autoimmunity [11].

**Table 1:** Enrichment *p*-values of antigen-presenting cell (APC) versus T cell interactions associated with type 1 diabetes.

APCs	T cells (E034)	T_h_ naive (E038)	T_h_ memory (E040)	T_reg_ (E044)
**Fetal thymus (E093)**	0.55	0.81	0.50	0.22
**Monocytes (E029)**	4.9 × 10^−4^	0.016	2.7 × 10^−4^	4.2 × 10^−6^
**B cells, PB (E032)**	8.4 × 10^−9^	1.4 × 10^−5^	4.4 × 10^−9^	2.7 × 10^−12^
**B cells, CB (E031)**	1.8 × 10^−5^	2.9 × 10^−3^	1.3 × 10^−5^	7.5 × 10^−8^

The *p*-values signify the over-representation of interacting single-nucleotide polymorphism pairs active in each cell type combination. See Fig 4B. Shown in parentheses are the epigenome codes. PB, peripheral blood; CB, cord blood.

**Fig 5:**
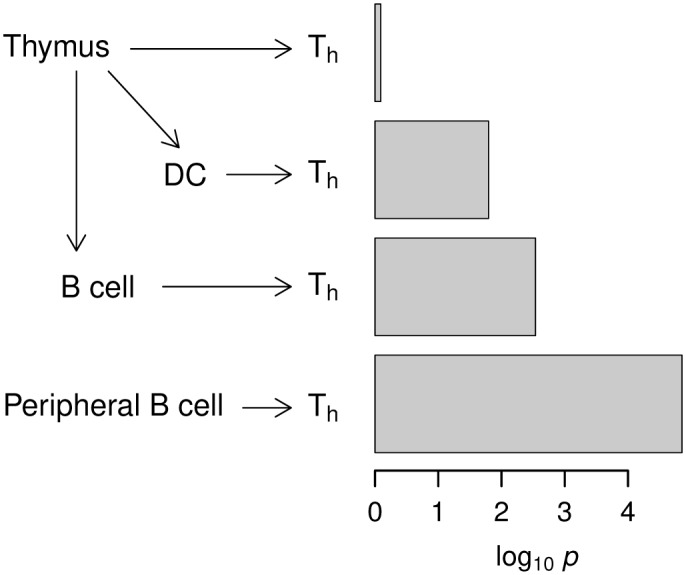
Sequence of relative importance of APC repertoire in T cell selection based on epigenomic enrichment. The *p*-values indicated (Table 1) refer to those of interactions with T_h_ naive for fetal thymus, monocytes, B cells (cord blood), and B cells (peripheral blood), respectively.

Based on the cell-type-specific interaction results in Table 1, we inferred the relative importance of different APC repertoire in autoimmunity (Fig 5) under the assumption that the epigenomes of naive T_h_ cells, fetal thymus, monocytes, and umbilical cord blood B cells are reasonable approximations of those for T cells in the thymus and periphery, mTECs, DCs, and thymic B cells, respectively. The sequence of significant enrichment patterns in Fig 5 indicates that direct interaction of T cells with thymus tissues is negligible, DCs presenting mTEC-derived self-antigens interact less strongly than B cells, and the interactions with peripheral B cells are strongest. The latter likely reflects the activation of cognate T_h_ cells that escaped the negative selection from the DC/B cell recognition in the thymus.

The interaction patterns shown in Fig 4B (T1D) additionally pointed to the interplay between lymphocytes and intestinal tissues. In particular, the interactions between B cells (E032/E031) and mucosal epigenomes (E101: rectum, E110: stomach, E077: duodenum, and E096: lung) reflect contributions from the MHC class III region near the *BTNL2* gene. BTNL2 belongs to the butyrophilin family of co-stimulatory proteins homologous to the B7 family (CD80 and CD86), the binding partner of CD28 (stimulatory) and CTLA4 (inhibitory) receptors on T cells [34]. BTNL2 is highly expressed in lymphocytes and intestinal epithelium cells (see Fig 2, *bottom*) and its simulation can inhibit T cell activation, in addition to inducing T_reg_ cell development (Fig 4C). Evidence for interactions between BTNL2 and B cells has also been reported [34]. One of the two T1D proxy SNPs (rs9268480) inside the coding region of *BTNL2* was 28 bp upstream from rs2076530, previously shown to cause splicing-related disruption of BTNL2 and an increased risk for sarcoidosis [35].

### Genetic interaction map

**Fig 6:**
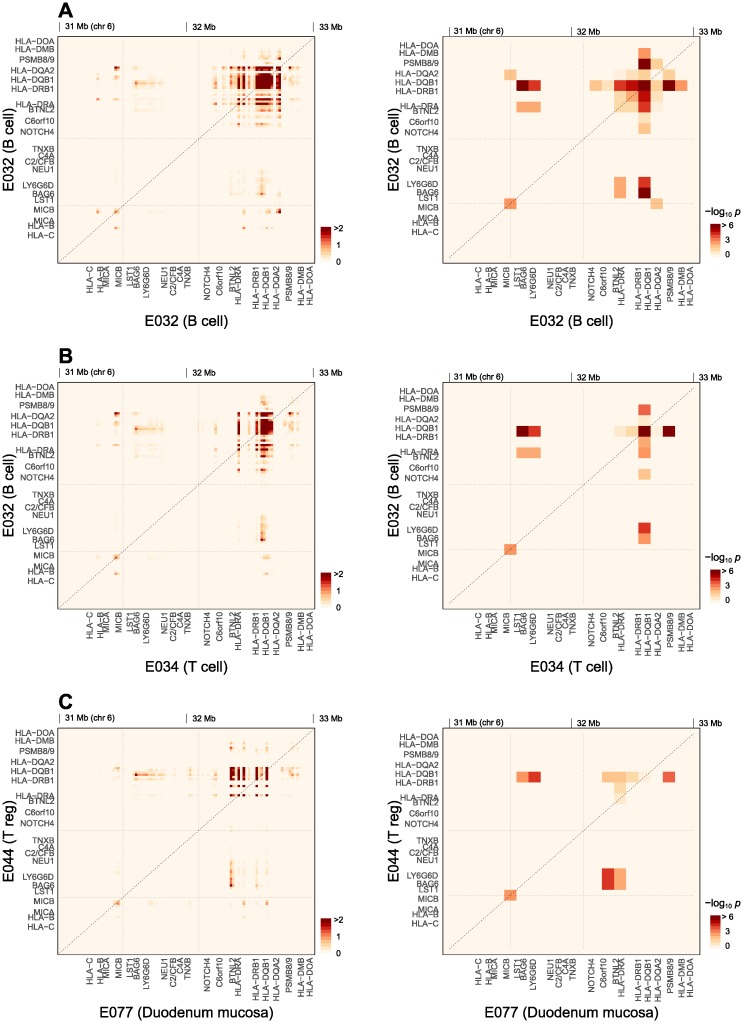
Spatial distribution of tissue-specific SNP-interactions for T1D. The overall enrichment profiles of cell type combinations in Fig 4B were resolved into contributions from genetic regions within the MHC locus. (**A**) B cell:B cell, (**B**) B cell:T cell, and (**C**) Duodenum mucosa:T_reg_ cell combinations. The left and right columns show the effective number of active SNP pairs (in a 20-kb grid) and the relative enrichment of this number within the given cell type combination against the total (in a 100-kb grid), respectively.

The interactions shown in Fig 4B count all contributions from SNP pairs active in each tissue combination. To test our second hypothesis regarding the spatial resolution of disease-associated interaction effects, we considered contributions from different genomic loci and generated histograms of significant SNP pairs active in specific cell types as functions of their genomic positions (Fig 6, *left*). For this analysis, we used T1D data that had higher effect sizes than RA. Non-MHC loci had only a few signatures of interactions with marginal significance (smallest *p* ~ 1×10^−3^) between *CTLA4*, *HLA-DR*, *HLA-DQ*, *IL2RA*, and *INS* loci (S1 Table). We focused on the MHC region in the subsequent analysis of genetic mapping. Since the frequencies of epigenetically active states at different genomic positions vary depending on tissues, we also obtained spatially resolved enrichment *p*-values (Fig 6, *right*) together with the pair distributions. The spatial resolution for the enrichment test was limited by the need to have sufficient numbers of interacting SNP pairs in each grid point. In this sense, the absolute (effective) pair number (*left* column) and enrichment *p*-values (*right* column) in Fig 6 each provide information regarding the genomic positions contributing to the interaction and their statistical significance, respectively. Three representative combinations of epigenomes are shown in Fig 6, with additional three in S6 Fig.

Globally, we grouped interactions into those within and between MHC class I (31–31.5 Mb), class III (31.5–32.1 Mb), and class II (32.1–33 Mb) regions. We first examined interactions within peripheral blood B cells (Fig 6A), which were dominant in Fig 4B. Broadly distributed interactions within and between *HLA-DQB1*, *HLA-DRB1*, and *HLA-DRA* genes were most notable. In professional APCs (B cells and DCs), the protein products of *HLA-DRA*, *HLA-DRB1*, *HLA-DQA1*, and *HLA-DQB1* form the MHC class II molecule in the ER, bind antigenic peptides, and present them on the cell surface to CD4^+^ T cells for recognition [31] (Fig 4C). The strong interactions within and between *HLA-DQ* and *DR* loci in B cells (Fig 6A) are consistent with this core component (antigen-presentation in thymic APCs) of T1D phenotype, and supports our earlier conclusion that thymic B cells are among the major components of the APC repertoire. The *HLA-DM* gene encodes a chaperone that facilitates the loading of peptides in the late endosome within this antigen presentation pathway [31, 36] (Fig 4C); hence, the interaction of the DM-proximal region with *HLA-DQ* and *DR*.

We also found evidence of interactions between *HLA-DQA2* and *HLA-DQB1* in B cells (Fig 6A). These two genes are paralogs of *HLA-DQA1* and *HLA-DQB1*, and are mostly silent, except in Langerhans cells [37]. Genetic associations of SNPs in the *HLA-DQA2/B2* locus have been observed in many other diseases. We noted above that the additive part of the association strength of *HLA-DQA2/B2* SNPs was reduced significantly in collective inference. The presence of interactions involving this locus shown in Fig 6A supports the notion that the effects of these genes on disease mechanism are cooperative in nature. We examined the epigenetic states of this segment of MHC class II locus (S5 Fig) and found them to be silent in most tissues, but *HLA-DQB2* was weakly transcribed in monocytes (E029) and B cells (E032), which is broadly consistent with the reported expression in Langerhans cells [37]. *HLA-DQB2* also contained a small (~0.4 kb) enhancer segment in the pancreas (E098). Importantly, *HLA-DQA2* contained a segment (~1 kb) of enhancers in B cells (E032) and hematopoietic stem cells (E051 and E036). We suggest that the interaction between *HLA-DQA2* and *HLA-DQB1* (Fig 6) indicates that the target genes of the B cell enhancer region in *HLA-DQA2* are likely *HLA-DQA1/B1* and *HLA-DR*.

Within the class III region, we observed significant interactions between the regions close to *BAG6* and *HLA-DQB1* as well as *HLA-DRA* genes. *BAG6* encodes a chaperone with a wide range of functions [38], one of which is the co-regulation of MHC class II expression in APCs together with *CIITA* [39]. Both CIITA and BAG6 are activated in response to interferon (IFN)-γ, which as a result stimulates APC function (Fig 4C). The *BAG6*-proximal region of the MHC class III locus was broadly expressed in all cell types (Fig 2). Its interaction with *HLA-DQB1* gene in Fig 6A is consistent with this MHC class II regulatory function of BAG6. In the class I region, there were signs of interactions within a region close to *MICB* gene and between *MICB* and *HLA-DR/DQ* loci.

We examined analogous interaction patterns between SNPs active in T cells and those active in B cells (Fig 6B). Some of the interactions observed in B cell self-interactions (Fig 6A) were weaker or absent in this combination. The presence of *HLA-DQB1* local interactions in Fig 6B likely reflects the fact that this region is also epigenetically active in T cells (Fig 2). Interactions involving *HLA-DQA2* and *HLA-DRA* from T cells were absent. On the other hand, the presence of interactions involving *BAG6*-proximal regions suggest a causal relationship: in addition to its regulatory function toward MHC class II molecules, *BAG6* is known to act as a negative regulator of *HAVCR2*, which is expressed in exhausted T cells along with CTLA4 [40] (Fig 4C). BAG6 rescues T_h_ cells from exhaustion and its overexpression can lead to increased risk for autoimmune diseases in mice [41].

Most notable in Fig 6B was the presence of interaction between the region close to *PSMB8/9* in T cells and the *HLA-DQ* locus in B cells (also present in B cell self-interactions in Fig 6A). *PSMB8/9* are located close to *TAP1/2* (S2 Fig) and Fig 6B per se does not make it clear which gene groups are causal. They each encode catalytic subunits of immunoproeasomes and ER peptide transporter complex, respectively, which together form an integral part of MHC class I antigen presentation pathway [31], whose relevance to autoimmunity, however, is likely to be secondary. Proteasomes also process key transcription factors, notably NF-κB [42], upon TCR activation, which allows for the expression of interleukin (IL)-2 and regulators such as FOXP3. In resting T cells, NF-κB remains in an inactive form as a part of inhibitors of κB kinase (IKK) complexes. When the peptide-MHC class II signal from APCs arrives via phosphorylation cascades, it allows for the polyubiquitination of the IKK complex. Degradation by the proteasome then releases the active NF-κB dimer, which enters the nucleus and binds to DNA to express key regulators leading to T cell proliferation and development (Fig 4C); IL-2 secretion and its recognition by IL-2 receptors encoded by *IL2RA/RB* are indispensable to T cell proliferation in general. FOXP3 is the distinguishing marker for T_reg_ cells, which, however, do not express IL-2 and rely on other CD4^+^ T cells for IL-2. A deficiency in β5i encoded by *PSMB8* can lead to autoimmunity [43] and affect the T cell lineage differentiation.

We therefore suggest that the interactions shown in Fig 6B reflect those between the polymorphisms in MHC class II molecules expressed in thymic B cells (and other APCs) and the immunoproteasome variants that affect TCR signaling. The two effects would each modify TCR activation signal strengths and NF-kB activation levels, which would in turn affect IL-2 and FOXP3 expression levels in negative selection and T_reg_ cell differentiation, respectively. Consistent with this interpretation, *PSMB8/9* versus *HLA-DQ* interactions in Fig 6B were significantly stronger when the two loci each belonged to T and B cells, respectively, compared to the B and T cell combination.

We also compared the interaction patterns between SNPs active in duodenum mucosa (representative of intestinal epithelial cells) and T_reg_ cells (Fig 6C), and confirmed our assertion that intestinal tissue interactions arose from *BTNL2* expressed in mucosal tissues, which can lead to T_reg_ cell development [34] (Fig 4C). Interestingly, the main interaction partner of epithelial *BTNL2* was the *BAG6* region in T_reg_ cells, which suggests that *BAG6* may also be involved in this process in addition to its interaction with HAVCR2. We found similar interaction signatures in the duodenum versus the B cell combination (S6 Fig), with stronger interactions between *BTNL2* and *HLA-DQB1*.

### Collective inference with pathway-based SNP selections

**Fig 7:**
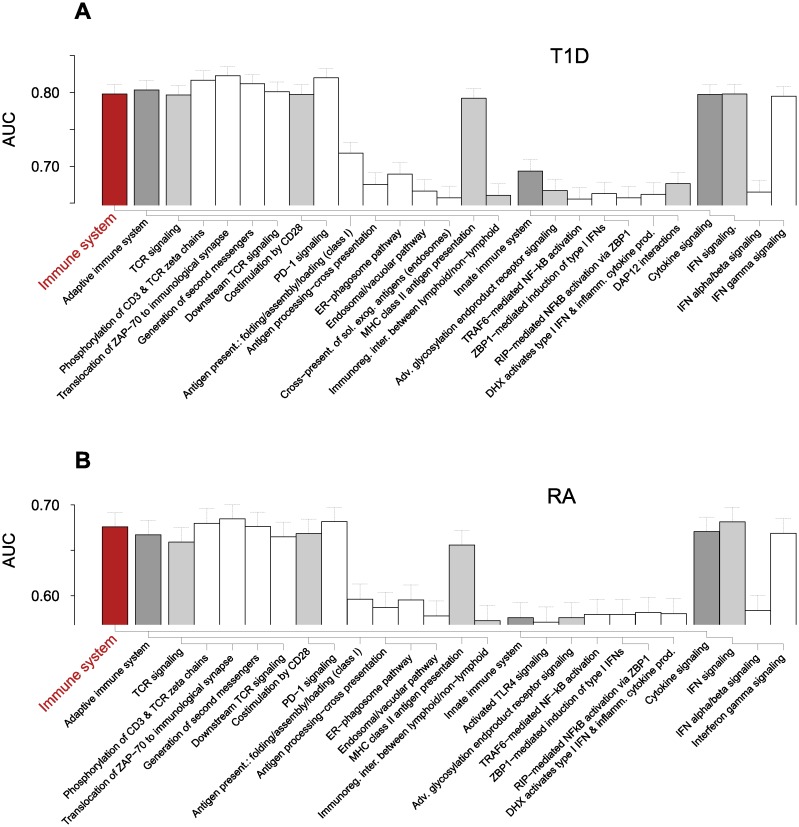
Immune system pathways scored by collective inference. The pathways belonging to the *Immune system pathway* in Reactome hierarchy in association with (**A**) T1D with AUC > 0.65 and (**B**) RA with AUC > 0.57 are shown. The dendrograms below the bars represent the relative hierarchical relationships. Error bars are 95% c.i. Adv., advanced; DAP12, DNAX activation protein of 12kDa; DHX, aspartate-glutamate-any amino acid aspartate/histidine (DExD/H) box helicase; ER, endoplasmic reticulum; exog. exogenous; immunoreg., immunoregulatory; IFN, interferon; inter., interation; PD-1, programmed cell death protein 1; present., presentation; RIP, receptor-interacting protein; sol., soluble; TCR, T cell receptor; ZBP1, Z-DNA binding protein 1.

To complement the analysis of interactions between the MHC loci, we selected genome-wide SNPs based on the Reactome pathways [44] and evaluated each SNP selection for association with autoimmunity. Our approach differs from other enrichment-based pathway analysis methods for SNPs [45] because all possible interaction effects between SNPs contained in each pathway are taken into account [27]. For T1D, the mean number of SNPs selected in each pathway ranged up to several thousands (excluding generic pathways at the top hierarchical level) and AUC scores up to ~0.80 (S7 Fig). Pathways containing genes within the MHC loci, including PSMB genes and *NOTCH4*, were highly scored, and most of non-immune pathways in T1D data with AUC > 0.6 contained PSMB genes. We therefore restricted our attention to the pathways belonging to *Immune system* group, aiming to gain insights into the effect of interactions involving MHC and other genetic loci. The 13 top-ranked pathways (AUC ~0.80 and ~0.68 for T1D and RA, respectively) were clearly separated from the rest and all consisted of MHC class II-related pathways (Fig 7 and S7 Fig). The analogous inference without interaction led to significantly lower AUC values (~0.75 and ~0.65 for T1D and RA, respectively; S7 Fig).

In Fig 7, the high association of *MHC class II antigen presentation*, represented by the class II genes including *HLA-DM* and *HLA-DO*, reflects the general dominance of *HLA-DQ* and *HLA-DR* loci in Fig 2: MHC class II molecules assembled in the ER with the invariant chain (Ii), initially occupying the peptide binding domain, moves into an endosomal compartment and Ii is exchanged with antigenic peptides with the help of HLA-DM and HLA-DO, which down-regulates HLA-DM in B cells [31, 36, 46]. The peptide-MHC class II complex is then displayed on the plasma membrane (Fig 4C). The subsequent recognition of the peptide-MHC class II complex by the TCR on CD4^+^ T cells and the resulting activation of T cells, represented by the *TCR signaling* pathways, showed similarly high association. The proximal part of *TCR signaling* after the MHC class II recognition (Fig 4C) starts with the recruitment of lymphocyte-specific protein tyrosine kinase (LCK) by CD4, which phosphorylates TCR complex and recruits ζ-chain associated protein kinase of 70kDa (ZAP-70) [47]. *PTPN22* downregulates this LCK action and thus TCR signaling, and its inhibition can lead to increased risks for autoimmunity [5]. These steps are described by *Phosphorylation of CD3 and TCR zeta chains* and *Translocation of ZAP-70 to immunological synapse*, to which both MHC class II genes and *PTPN22* contribute. ZAP-70 leads to the formation of linker for activation of T cells (LAT) complex comprising a host of binding partners that include adhesion and degranulation-promoting adaptor protein (ADAP) and phospholipase C (PLC)-γ. The LAT complex leads to three major downstream pathways: the Ca^2+^, the mitogen-activated protein kinase (MAPK) kinase and the NF-κB signaling pathways, in addition to the “inside-out” signaling that activates integrins via actin cytoskeleton reorganization (Fig 4C). The inside-out pathway is mediated by the binding of ADAP to Drosophila enabled (Ena)/vasodilator-stimulated phosphoprotein (VASP), a regulator of actin polymerization [48], and results in the formation of activation clusters centered around peptide-MHC-TCR complexes allowing for enhanced interactions between APCs and T cells [49]. In addition to MHC class II genes, the pathway *Generation of second messenger molecules* was implicated by *ENAH* that encodes the Ena protein. *ENAH* polymorphisms may thus affect the integrin-mediated APC-T cell adhesion component of the TCR activation process: there were six associated SNPs with *p*_i_ < 10^−3^ in the coding region of *ENAH* (S8 Fig) in T1D with the strongest association by rs2639703 (*p*_*i*_ = 1.2×10^−5^).

*Downstream TCR signaling* represents the process leading to the activation of transcription factor NF-κB, which enters the nucleus and allows for the expression of many regulators and cytokines, including IL-2 and FOXP3 (Fig 4C), thus playing critical roles in T_reg_ cell development [42]. This pathway contains *NFKB1*, *CDC34*, *PSMB8/9*, and *PSMD14*, in addition to MHC class II. *NFKB1* encodes the p105 subunit of the NF-κB protein family, which undergoes proteasomal processing upon TCR activation and combines with a p65 subunit to form the active NF-κB heterodimer [42] (Fig 4C). The association of *NFKB1* indicates that the NF-κB downstream pathway is central to autoimmunity [1] and the gene was represented by rs1314336 (*p*_*i*_ = 6.0×10^−4^, 44 kb upstream; S8 Fig). The disease association of proteasome-encoding genes and *CDC34* in this pathway reinforces this interpretation: IKK undergoes TCR signal-induced polyubiquitination, a prerequisite for proteasomal degradation necessary to produce a functional NF-κB molecule. The *CDC34* gene encodes the E2 enzyme catalyzing ubiquitination [50], represented by rs4919910 located 22 kb upstream (*p*_*i*_ = 1.6×10^−4^;S8 Fig). The *PSMB8/9* region in the MHC class II locus had 19 SNPs implicated (top association: rs241427, *p*_*i*_ = 4.75×10^−62^). *PSMD14* encodes a subunit in the 19S regulatory cap complex and had moderate levels of association (*p*_*i*_ ~ 10^−3^; S8 Fig).

*Costimulation by the CD28 family* contains co-stimulatory and co-inhibitory signaling pathways that reinforce or downregulate TCR signaling [51] and contains *CTLA4* and *PTPN11* in addition to MHC class II. CD28 co-receptors bind CD80/CD86 ligands expressed on APC surfaces and upregulate proximal signaling. CTLA4 competes for CD80/CD86 binding and, along with PD1, recruits phosphatases including PTPN11, which dephosphorylates key signaling complexes (Fig 4C). The class I antigen presentation and cross-presentation pathways had significantly lower AUCs. The MHC class I pathways can affect autoimmunity via the CD8^+^ T cell selection in the thymus and also during late-stage pathogenesis during effector T cell activation, which, however, would involve constitutive proteasomes rather than immunoproteasomes. CDC34 is included in these pathways as a candidate E2 enzyme that may substitute UBE2A. SOCS1 mainly functions as a negative regulator of cytokine signaling, but can also participate in E2-mediated ubiquitination [52]. TRIM39 is one of the RING domain-containing proteins that share broad activities as E3 ubiquitin ligases [53]. The multiple appearances of these proteasome-related genes in different pathways suggest that proteasomes play key roles in the control of autoimmunity. Overall, the relatively lower association strengths of these pathways imply that autoimmune risk is mainly driven by CD4^+^ T cell (T_h_ and T_reg_) selection and activation involving MHC class II molecules.

The *Cytokine signaling* pathway group was among the top-ranked pathways, primarily due to *Interferon gamma signaling*, which describes the classical Janus kinase (JAK)-signal transducer and activator of transcription (STAT) pathway initiated by the binding of IFN-γ to its receptor that leads to the expression of numerous genes [54], including MHC class I/II and *CIITA*, which controls class II expression (Fig 4C). The MHC class I and II genes present together in this pathway as downstream targets explain its high AUC. *SOCS1*, *PTPN11*, and *PTPN2* [55] encode proteins that inhibit JAKs and act as negative regulators. IFN-γ also induces the expression of a large number of TRIM genes, many of which act as E3 ubiquitin ligases for proteasomal degradation [53]. Three TRIM proteins (TRIM10/26/31), located in the MHC region (S8 Fig), appeared as the target genes of this pathway, supporting the autoimmunity association of proteasomes. *IL-2 signaling*, containing the key disease-associated genes *IL2* and *IL2RA/IL2RB* (encoding receptor α and β chains), had AUC = 0.62 in T1D. These genes accounted for 10 SNPs with *p*_*i*_ < 10^−3^ (S8 Fig). The binding of IL-2 on IL-2R activates *JAK1* and *JAK3* and lead to multiple signaling pathways, including Ras-MAPK, JAK-STAT, and phosphoinositol (PI)3-kinase pathways. The Ras-MAPK pathway is activated by the recruitment of SH2 containing (SHC) protein by the IL-2/IL-2R complex. *RAF1* and *BRAP* negatively regulate Ras-MAPK pathway, the latter using ubiquitination/proteasomal degradation. *BRAP* is located close to *PTPN11* and they appeared to form a loci of association together on chromosome 12 (S8 Fig). Among many other input signals to the Ras-MAPK pathway is *ERBB3*, one of the established T1D-associated loci [10]. ERBB3 forms the starting point of the epidermal growth factor receptor kinase signaling involving the Ras-MAPK pathway, which can influence the gene expression in APCs (Fig 4C).

### Association of pathway-implicated genes in larger data sets

We compared the association of non-MHC genetic regions contributing to pathways in the RA data (S8 Fig) and found generally weaker association than in T1D, except in *PTPN22*, where the RA association was equally strong and in *IL2RA* (rs10795791 and rs2104286) and *IL2RB* regions (rs3218253 and rs743777), where there were stronger associations. To further evaluate the significance of these genetic regions, we compared them based on the summary statistics of two large-sample T1D data sets by Bradfield et al. (BRF) [8] and Barrett et al. (BRT) [10] (S9 Fig). Some of these genes were within/close to previously identified loci: *PTPN22*, *CTLA4*, and *IL2RA* are among the major robustly identified autoimmune risk loci [6] and all showed higher degrees of association in the BRF/BRT data sets. In addition, we noted significant groups of SNPs in the BRF data set near *CD28* upstream from *CTLA4*; in TCR signaling, *CD28* and *CTLA4* each act as stimulatory and inhibitory co-receptors, respectively (Fig 4C). *PSMD14*, which had a number of SNPs with *p*_*i*_ ~ 10^−3^ in T1D, was surrounded by a large number of SNPs but with similarly low levels of significance in the BFR/BFT data sets, while being located close to the previously identified *IFIH1* locus [6]. *IL2* at 4q27 is a part of the locus centered around *KIAA1109* and showed increased levels of significance in larger data sets. *ERBB3* and *PTPN2*, also identified in previous studies [6], showed increased levels of significance in larger data sets (S9 Fig). Along with *ERBB3* at 12q13, *BRAP* and *PTPN11* at 12q24 form the two main regions of the chr-12 loci (S1 Fig). This broad region of association had increased significance levels in the larger studies with the *BRAP*-proximal region reaching *p*_*i*_ ~ 10^−30^ in the BRT data set. *IL2RB* is within the *C1QTNF6* locus in 22q12, which showed a moderate increase in association strength. The *CLEC16A* locus on chromosome 16 contained *CIITA* and *SOCS1*.

The *ENAH*, *RAF1*, *NFKB1*, and *CDC34*-proximal loci are regions with potential association of collective origin; when we compared their association patterns in WTCCC and larger data sets (S8 and S9 Figs), they either showed a moderate increase in significance in the larger studies or instead had similar *p*_*i*_ ranges but showed more well-defined appearance of a peak centered around the genes.

## Discussion

The cell-type-specific interactions between genetic polymorphisms we characterized illuminate two different aspects of autoimmune disease pathogenesis: central and peripheral tolerance. The central tolerance in the thymus comprises both negative selection and T_reg_ development, whereas peripheral tolerance involves the suppressive action of thymic T_reg_ (tT_reg_) cells originating from the thymus as well as the peripherally induced T_reg_ (pT_reg_) cell development from naive FOXP3^-^CD4^+^ T cells [56]. The strength of self-interactions between thymocyte TCRs and self-peptide-MHC class II complexes presented by APCs (mTECs, DCs, and thymic B cells) is the core determinant influencing both the negative selection and the development of tT_reg_ cells: intermediate and high levels of avidities likely favor tT_reg_ differentiation and negative selection, respectively [22, 57].

mTECs express and present TRAs induced by AIRE or transfer them to DCs, whose migratory variants can also bring in peripheral antigens for presentation. Recent studies have indicated that thymic B cells, either developed within or having immigrated from the periphery, can also efficiently present self-peptides to immature T cells [19, 20], facilitating negative selection as well as T_reg_ cell development [58]. Yamano et al. showed that this conversion of peripheral B cells requires a CD40 signal and that thymic B cells also express TRAs modulated by AIRE [20]. Peripheral B cells, while generally inefficient as APCs, can present BCR-recognized peptides and activate cognate CD4^+^ T cells. B cells have been found to present pancreatic β-cell autoantibodies to T cells and contribute to T1D [14]. We found predominantly strong disease-associated interactions between B cells/DCs and T cells, whereas the corresponding interactions were minimal for mTECs (Fig 4 and Table 1), which support the experimental studies suggesting central roles played by thymic and peripheral B cells as APCs. It has been suggested, in particular, that AIRE-induced TRA expression profiles in B cells and mTECs are likely to be distinct, and that the APC function of thymic B cells may serve to influence CD4^+^ T cell populations such that BCR-induced activation of autoimmunity in the peripheral tissues could be minimized [20]. Our results imply that the MHC class II polymorphisms exert their dominant effects on autoimmune risk primarily via their presentation on thymic B cells. Impairment of this B cell-specific negative selection and T_reg_ development are consistent with the widespread detection of autoantibodies in late-stage T1D pathogenesis [11].

Although both negative selection and T_reg_ development result from TCR signaling, with their respective fates likely determined by relative MHC-TCR recognition strengths, there are distinct molecular level features characterizing T_reg_ development, notably FOXP3 expression. In particular, among the possible products of downstream TCR signaling, evidence indicates that NF-κB is the primary transcription factor crucial for T_reg_ cells: mice lacking its main signaling intermediates (PKCθ, CARD11, and IKK; see Fig 4C) develop severe T_reg_ deficiency [22]. Our results support the relative importance of thymic B cell-T_reg_ interactions during T cell maturation (Table 1) and the central importance of NF-κB signaling (Fig 7).

It is further believed that T_reg_ cell differentiation consists of two distinct steps: the first step produces a FOXP3^-^CD25^+^ T_reg_ cell precursor as a result of MHC-TCR interaction and NF-κB binding to a *FOXP3* enhancer element. The TCR-independent second step requires the binding of IL-2 (expressed by other CD4^+^ T cells) to the (TCR signaling-induced) IL2 receptor, which produces STAT5 that stimulates the expression of FOXP3 (Fig 4C) [22]. SNPs outside the MHC region influencing these pathways have association strengths much weaker than MHC loci. Through pathway-based SNP selection combined with collective inference, we correctly ranked these key pathways (Fig 7) and identified relevant genetic factors, some of which were among the set of well-characterized loci (*CTLA4*, *PTPN22*, and *IL2RA*), while others were close to previously identified loci (*IL2*, *CIITA/SOCS1*, and *IL2RB*), helping to illuminate their biological interpretations. Some were those with levels of association below genome-wide significance (e.g., *ENAH*, *RAF1*, *NFKB1*, and *CDC34*) and, individually, would look unremarkable within a genome-wide scan, but collectively contributed to the overall disease association of pathways such as *IL-2 signaling*, which is devoid of MHC SNPs.

Our analysis also pointed to two factors whose roles in autoimmunity have been suggested experimentally but their genetic contexts have not been clear. One is *BAG6*, whose multiple appearances in B cell self-interactions (Fig 6A) and B cell-T cell interactions (Fig 6B) are readily explained by its dual roles in MHC class II expression in APCs and the negative regulation of HAVCR2 (and FOXP3) in T cells (Fig 4C). The other, *BTNL2* expressed on intestinal epithelial cells, explains a different aspect of peripheral tolerance via its interaction with T_reg_ cells (Fig 6C): the induction and maintenance of pT_reg_ cell populations in intestines by microbial and self-antigens [34]. pTreg cells appear to have TCR specificities distinct from tTregs and presumably act cooperatively with tTregs to suppress self-reactive effector T cell activation [56]. Based on our observation of interactions involving intestinal epithelial cells in Fig 4B, we suggest that both DCs (by presenting antigens derived from intestinal cells) and B cells (by BCR-induced antigen presentation) contribute to pT_reg_ development in the gut. This peripheral tolerance component involving pT_reg_ cells also likely explains the effect of environmental factors on T1D risk: non-obese diabetic mice are less prone to T1D when exposed to microbes, and the human disease is more prevalent in industrialized societies with limited exposure to bacteria [11]. The large proportion of effector B and T cells in intestinal tracts also implies that the activation of conventional T_h_ cells cognate to self-antigens (presented for example by migratory DCs) may occur in or near intestinal organs, whose action toward effector cells is suppressed by tT_reg_ and pT_reg_ cells.

It is worth emphasizing that the main results discussed above arise from effects beyond single-SNP association: Fig 4A, for instance, cannot resolve cell-to-cell interactions. This cooperative nature of genetic interactions is further illustrated by *BAG6* and *BTNL2*, whose variants had significantly reduced association in the additive component of *p*-values (Figs 2 and 3) under collective inference, featuring strongly instead in interaction maps (Fig 6). This finding is consistent with the expectation that because SNPs near *BAG6* affect disease by modifying CIITA-based regulation of MHC class II antigen-presentation pathway [39] (Fig 4C), they are dependent on *HLA-DQB1* variants. Likewise, SNPs in the *BTNL2* region presumably affect the affinity of the ligand expressed on the surface of APCs toward a receptor yet to be identified on T cells, contributing to T_reg_ cell development [34]. In particular, Fig 6C suggests that in addition to negatively regulating HAVCR2 signaling [41], BAG6 may also act on the signaling of this BTNL2 receptor (Fig 4C).

We also noted interactions involving *MICB* in the class I region in Fig 6. *MICB* and neighboring *MICA* encode molecules highly expressed in intestinal epithelium (rs2248459 and rs2248617 in Fig 2) and are recognized by the similarly localized Vδ1 subset of γδ T cells [59]. In this context, γδ T cells in intestinal tissues help eliminate foreign antigens and stressed cells, which may also contribute to the control of autoimmunity.

Our computational analysis adopted a shift in spirit from other studies testing for possible interactions between SNPs: instead of aiming to find a few causal interaction pairs among many with a sufficiently small false discovery rate, we established a large pool of interacting pairs with a liberal significance threshold and derived coarse-grained features, namely the cell type-specific interaction patterns in Fig 4B. The low-dimensional nature of the derived features supports such analysis. For instance, ~100 potentially significant interactions contributing to such features would not be affected significantly even when false positives among them number more than ~5. We anticipate that applications of similar approaches to other disease classes based on large-scale, meta-analysis data may also yield useful biological insights.

## Methods

### Analysis software and computations

We used GeDI (genotype distribution-based inference) [27], available at http://www.github.com/BHSAI/GeDI, for independent-SNP analysis, collective inference, cross-validation, and interaction *p*-value calculations. The genotypic model (2 and 4 degrees of freedom for additive and interaction parts, respectively) was used for all cases. For data management tasks and LD calculation, we used PLINK [60] version 1.9. We verified that non-interacting *p*_*i*_-values from GeDI were similar to the PLINK outputs. For consistency with collective inference results, we used the GeDI results for the special cases of independent-SNPs.

For collective inference, we used the pseudo-likelihood method with *l*_2_ penalizer λ, except for pathway scoring, for which we used the mean-field option. Computational costs for the former were of the order of ~1 hr for a single collective inference involving ~100 SNPs on a desktop machine without additional tests. We performed interaction *p*-value calculations (based on phenotype label permutation) using the Department of Defense High-Performance Computing resources: for *m* = 100 SNPs, this calculation would consider 100×99/2 = 4,950 SNP pairs, each of which was repeated at least 3,000 times to construct the null distributions, amounting to ~10^7^ h if run on a single desktop computer.

### Genome-wide data processing

We obtained the WTCCC case-control data for T1D and RA and built the case and control data sets excluding individuals as specified in the quality control report of the original study [6]. The resulting samples contained 2,938 control individuals (shared), 1,963 T1D case individuals and 1,860 RA case individuals. With the quality-controlled matrix-format genotype data provided, we first performed a preliminary association analysis and identified SNPs with *p*_*i*_ < 10^−3^ for each disease. We then visually inspected the distribution of bi-allelic raw signals underlying the genotype calls for each SNP and excluded variants with poor clustering as previously described [6]. We further excluded isolated SNPs that showed strong association strengths without neighboring variants and obtained 458,934 and 458,511 SNPs for T1D and RA, respectively. All genomic positions are with respect to the GRCh37 reference.

### Cross-validation

We performed five-fold cross-validation using the genome-wide data under varying values of an *l*_2_ penalizer λ by first selecting a cutoff *p*_*c*_ and selecting SNPs with *p*_*i*_ < *p*_*c*_ using the training set only. The number of SNPs selected in this way varied with each cross-validation run because the training sets were distinct. For each selection, we inferred both additive and interaction parameters involving the selected SNPs using maximum likelihood and predicted disease phenotypes for the test set based on the disease probability given by Bayes’ theorem. These predictions from all cross-validation runs were combined to calculate the AUC. These steps were repeated for different λ values and *p*_*c*_ values. We calculated the mean values of the number of SNPs selected for each choice of *p*_*c*_.

### Collective inference

For T1D analysis, we selected 50 SNPs from chromosome 6 based on *p*_*i*_ value ranks, and 10 SNPs each from *PTPN22*, *CTLA4*, *INS*, *IL2RA*, and chr-12 loci to form the *m* = 100 SNP set. Additive and interaction parameters for this SNP selection were inferred under the penalizer value λ = 0.01 suggested by the cross-validation AUC maximum. The *p*-values associated with the additive single-SNP parameters (Figs 2 and 3, solid bars, *middle*) were obtained by the asymptotic *χ*^2^ null-distribution approximation. For RA, we first selected 255 SNPs in chromosome 6 (MHC region) from the genome-wide data using *p*_*c*_ = 10^−4^. We then calculated LD within this selection of SNPs and used the negative logarithm of LD *r*^2^ values as a distance measure to cluster them (*k*-means clustering) into *m* = 70 clusters, each with high LD within the group. From each group, we then selected a SNP with the lowest *p*_*i*_ value to form the final *m* = 70 proxy SNP selection. We used λ = 0.01 for collective inference of these RA data, again based on the cross-validation AUC maximum.

Interaction *p*-values were obtained using methods as described [27]. Briefly, we first calculated likelihood ratio statistics corresponding to the alternative hypothesis of the full optimization of each SNP pair interaction versus the null hypothesis of the SNP interaction parameters being identical for both case and control groups. Null distributions of these statistics for each interaction were obtained by repeating the collective inference after randomly shuffling phenotype labels. This sampling was repeated at least 5,000 times for T1D and 3,000 times for RA. As sampling depths increased, *p*-value estimates for putatively significant pairs tended to increase. The final sampling depths were such that this convergence reached reasonable saturation.

### Epigenetic enrichment analysis

We used genome-wide annotations of epigenetic states in 111 reference epigenomes based on the 15-state hidden Markov model [32], reducing them into *active* (transcribed: TssA, TssAFlnk, TxFlnk, Tx, TxWk; enhancer: Enh, EnhG, and zinc finger-associated: ZNF, Rpts) and *inactive* (heterochromatin: Het, bivalent: TssBiv, BivFlnk,EnhBiv, repressed: ReprPC, ReprPCWk; quiescent: Quies) states. We obtained the frequency of active states in each epigenome by calculating the fraction of genomic segments in active state. For the proxy SNP sets of each diseases (T1D and RA), we constructed the group of all known SNPs in LD with each proxy SNP (*r*^2^ > 0.5) from 1000 Genomes Project Phase 3 data [30]. The size of these “LDed” SNP groups ranged from 1 (proxy alone) to ~300 or more for each proxy. We calculated the mean active-state frequencies of each proxy (Figs 2 and 3, *bottom*) in different epigenomes over these LDed SNP groups.

The enrichment *p*-values in reference epigenomes on the single-SNP level (Fig 4A) were obtained by comparing these frequencies with the background genome-wide frequencies. We used binomial tests by multiplying the frequencies by the total number of SNPs (*m* = 100 and 70 for T1D and RA, respectively) with fractional numbers rounded up to integers. For the enrichment of interacting SNP pairs, we first selected SNP pairs with interaction *p*-values below a cutoff (*p* < 10^−3^). We then considered all possible combinations of two epigenomes, and counted the number of LDed SNP pairs belonging to the selected proxy SNP pairs where both LDed SNPs were active in each respective cell types. This number was divided by the product of the two LDed SNP group sizes and summed over the proxy set to obtain the effective number of active-state pairs. This number was compared using binomial tests with the background value estimated by the product of active state frequencies in two tissues and the total number of significant SNP pairs.

To obtain spatially resolved genetic interaction maps (Fig 6), we divided the MHC region (31–33 Mb on chromosome 6) into 20 kb-sized bins and built two-dimensional histograms of the LDed SNP pairs belonging to each grid active in two chosen tissue combinations, normalized by the product of LDed group sizes such that the numbers would represent the effective number of active SNP pairs. To estimate the spatially resolved enrichment *p*-values, we enlarged the grid size to 100 kb such that an appreciable subset of grids would have effective SNP pair counts larger than 1. We then compared these effective numbers in each grid points with the epigenome-wide average using binomial tests.

### Collective inference with pathway-based SNP selection

We downloaded human genomic pathway (1,705 in total) gene lists from www.reactome.org on April 19, 2016 and made a list of all non-provisional genes involved in the pathways. From all quality-controlled sets of SNPs, those within the distance of 50 kb from the coding region were assigned to each gene. We then represented each pathway by the non-redundant list of SNPs assigned to the constituent genes. The number of SNPs selected in each pathway ranged up to ~10,000 (S7 Fig). For each pathway, we used independent-SNP and collective inferences (mean-field approximation [27]) to find AUC scores. For largest pathways with more than ~2,000 SNPs, *p*_*i*_ value-based filtering was applied to cross-validation to reduce model sizes. We downloaded the summary statistics data of the T1D meta-analyses by Bradfield et al. [8] and Barrett et al. [10] from www.t1dbase.org to generate the genetic association map of the loci suggested by pathway analysis (S9 Fig).

## Supporting Information

S1 Fig Coverage of SNPs in LD with T1D proxy SNPs. Positions of 1000 Genomes Project SNPs with LD (*r*^2^ > 0.5) to *m* = 100 T1D proxy SNPs (Fig 2) are shown with proximal gene coding regions at the bottom. Generated with the University of California, Santa Cruz (UCSC) Genome Brower, https://genome.ucsc.edu.
(PDF)

S2 Fig Detailed view of the SNP coverage in LD with the four T1D proxy SNPs near the *PSMB8/9* genes. See S1 Fig.
(PDF)

S3 Fig LD pattern between T1D proxy SNPs. See Fig 2 for the approximate gene annotations of the proxy SNPs.
(PDF)

S4 Fig Coverage of SNPs in LD with RA proxy SNPs. Positions of 1000 Genomes Project SNPs with LD (*r*^2^ > 0.5) to *m* = 70 RA proxy SNPs (Fig 3) are shown with proximal gene coding regions at the bottom. Generated with the UCSC Genome Browser, https://genome.ucsc.edu.
(PDF)

S5 Fig Epigenetic annotation of the genomic region near *HLA-DQA2* and *HLA-DQB2* genes. The map was generated using the Roadmap epigenome browser at http://epigenomegateway.wustl.edu/browser.
(PDF)

S6 Fig Spatial interaction map of T1D-associated interacting SNPs enriched in three cell type combinations. (PDF)

S7 Fig Distribution of pathway association scores with T1D and RA phenotypes. Inferences with and without interaction effects are shown together as functions of the number SNPs in each pathway. Pathways containing MHC class II genes are shown in red. Vertical lines are 95% c.i. IL, independent loci inference; CL, collective loci inference.
(PDF)

S8 Fig Independent-SNP *p*-value profiles of pathway-implicated genetic regions for T1D/RA. See Fig 4C. Genes shown in red are those in top-ranked pathways in Fig 7, whose coding regions are shown shaded in gray. Other genes of interest nearby are indicated with non-shaded coding regions. Darker blue (T1D) and orange (RA) symbols are the SNPs directly included in the pathways (within 50 kb of coding region and *p*_*i*_ < 10^−3^).
(PDF)

S9 Fig Independent-SNP *p*-value profiles of pathway-implicated genetic regions based on large T1D meta-analyses. Data are from summary statistics of studies by Bradfield et al. [8] and Barrett et al. [10]. See S8 Fig for comparison.
(PDF)

S1 Table Inter-loci interactions between T1D proxy SNPs with low *p*-values. (XLS)
