# Supplementary figures and images for: Collective Genetic Interaction Effects and the Role of Antigen-Presenting Cells in Autoimmune Diseases
Source: PLoS One. 2017 Jan 12;12(1):e0169918. doi: 10.1371/journal.pone.0169918 (PMC5231276; doi:10.1371/journal.pone.0169918)

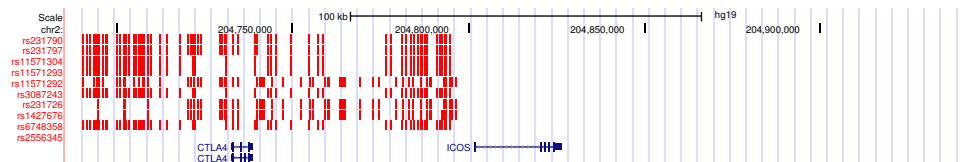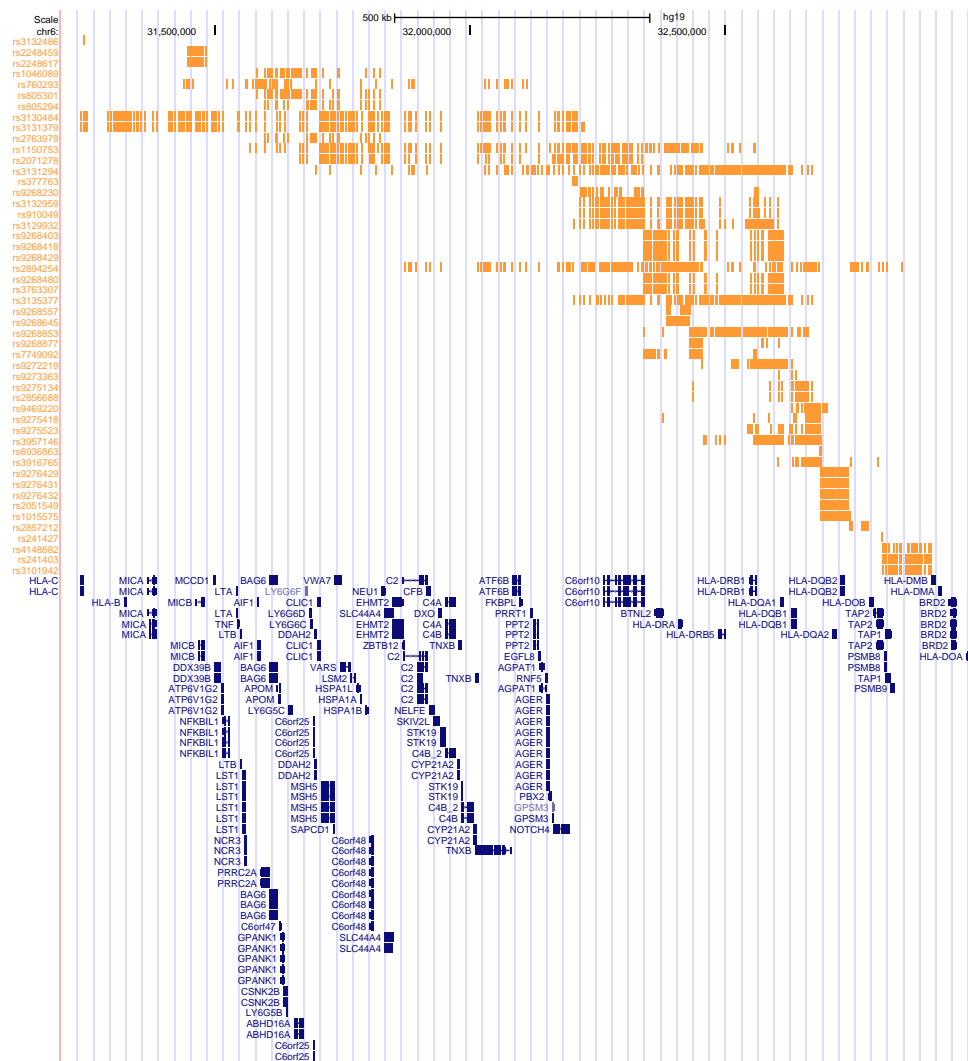

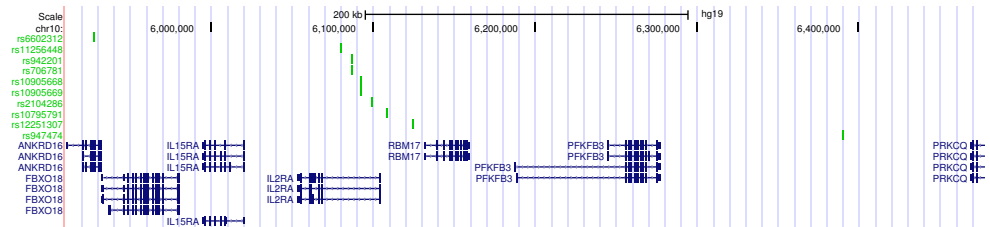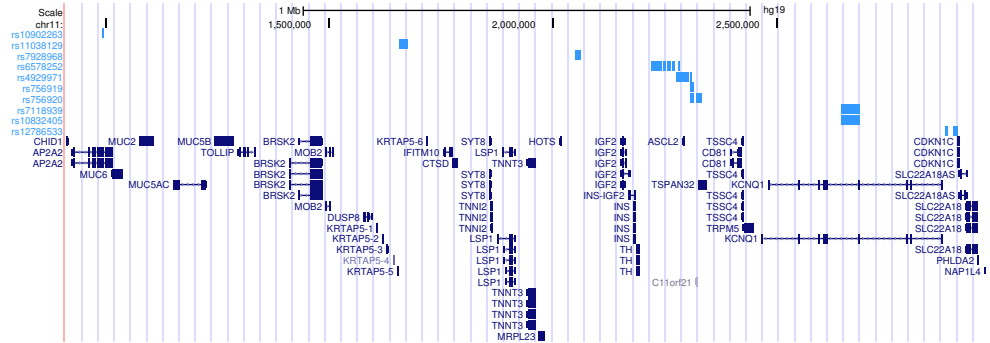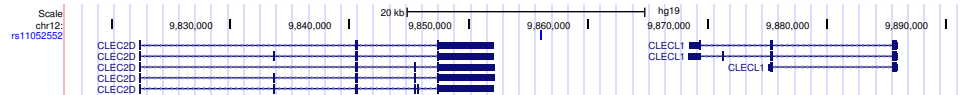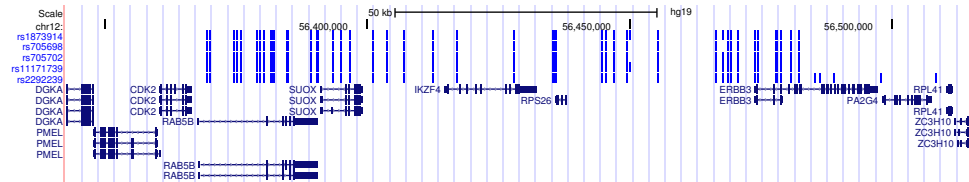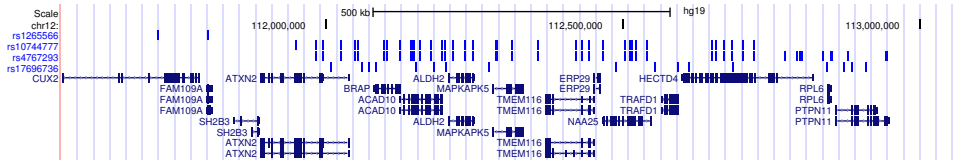

Supplement: S1 Fig — Positions of 1000 Genomes Project SNPs with LD (r2 > 0.5) to m = 100 T1D proxy SNPs (Fig 2) are shown with proximal gene coding regions at the bottom. Generated with the University of California, Santa Cruz (UCSC) Genome Brower, https://genome.ucsc.edu. (PDF) [file pone.0169918.s001.pdf]

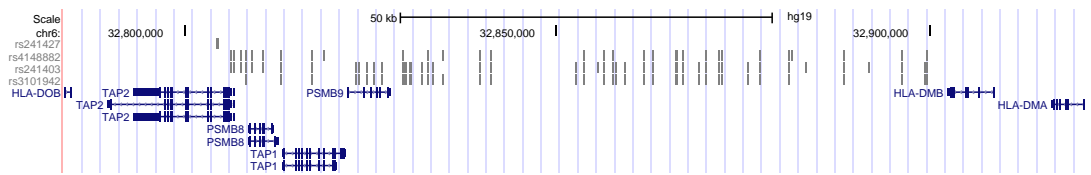

Supplement: S2 Fig — See S1 Fig. (PDF) [file pone.0169918.s002.pdf]

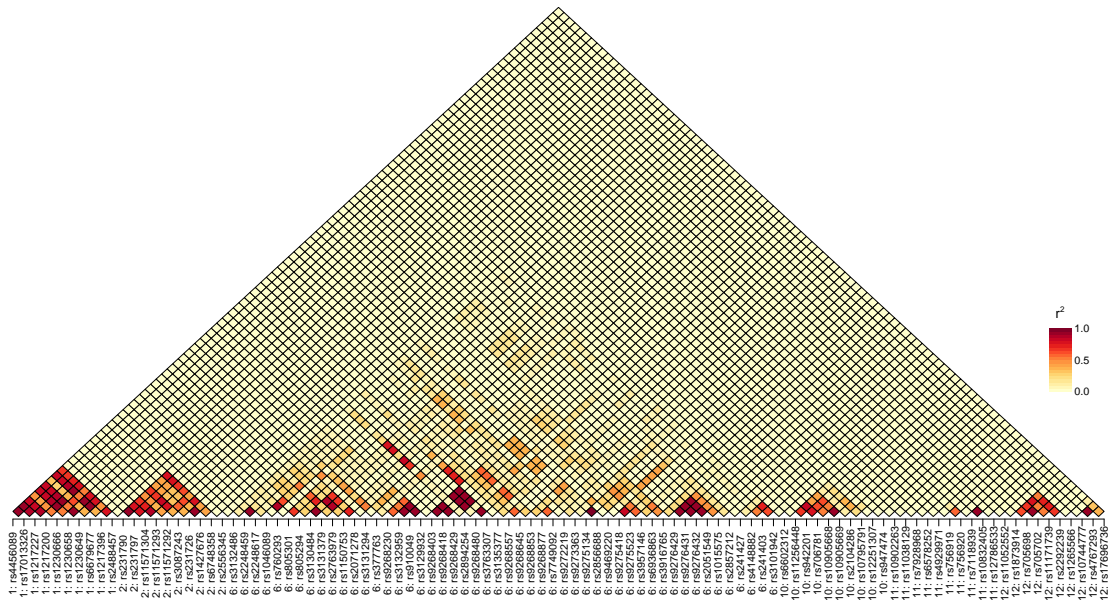

Supplement: S3 Fig — See Fig 2 for the approximate gene annotations of the proxy SNPs. (PDF) [file pone.0169918.s003.pdf]

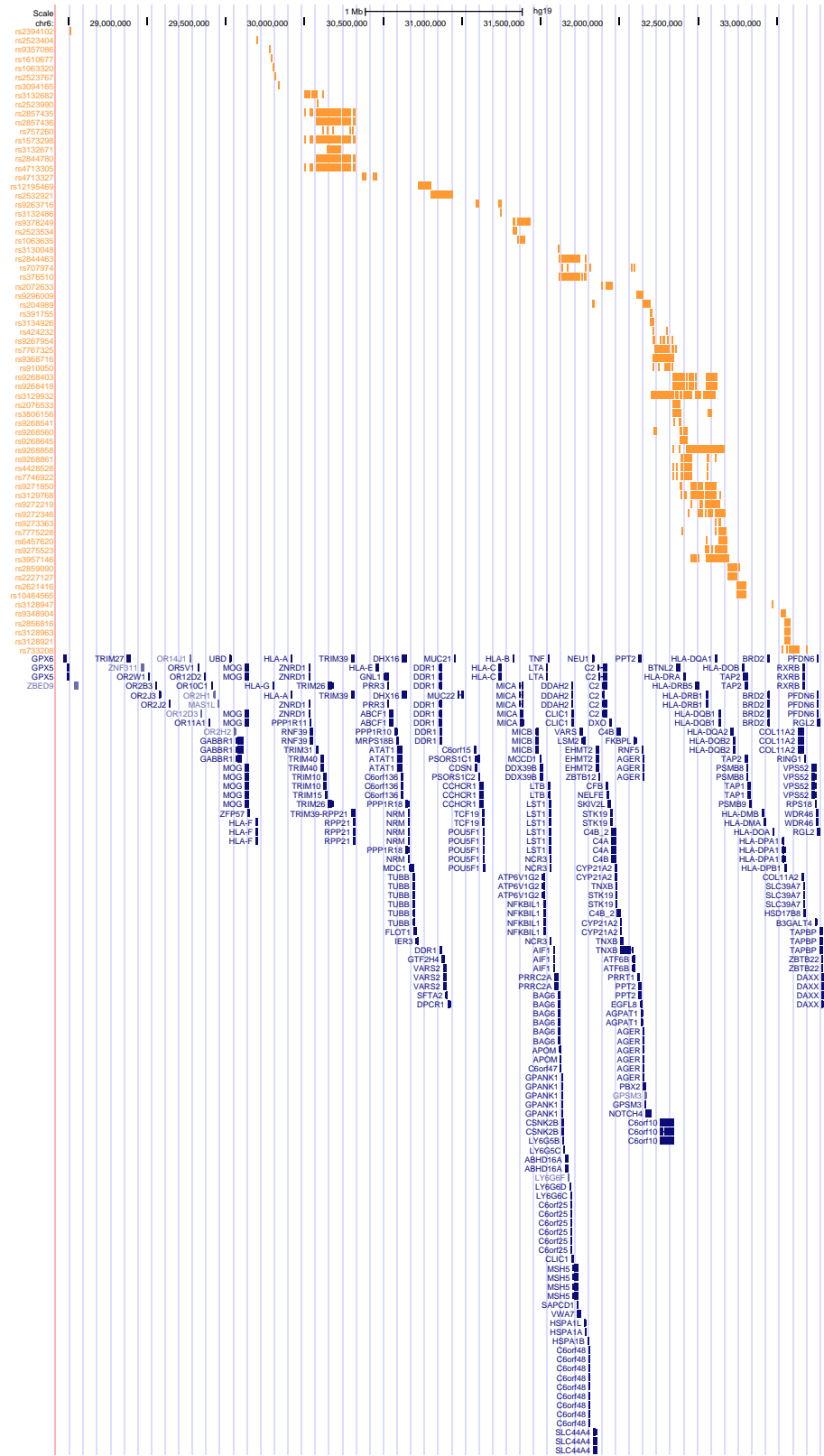

Supplement: S4 Fig — Positions of 1000 Genomes Project SNPs with LD (r2 > 0.5) to m = 70 RA proxy SNPs (Fig 3) are shown with proximal gene coding regions at the bottom. Generated with the UCSC Genome Browser, https://genome.ucsc.edu. (PDF) [file pone.0169918.s004.pdf]

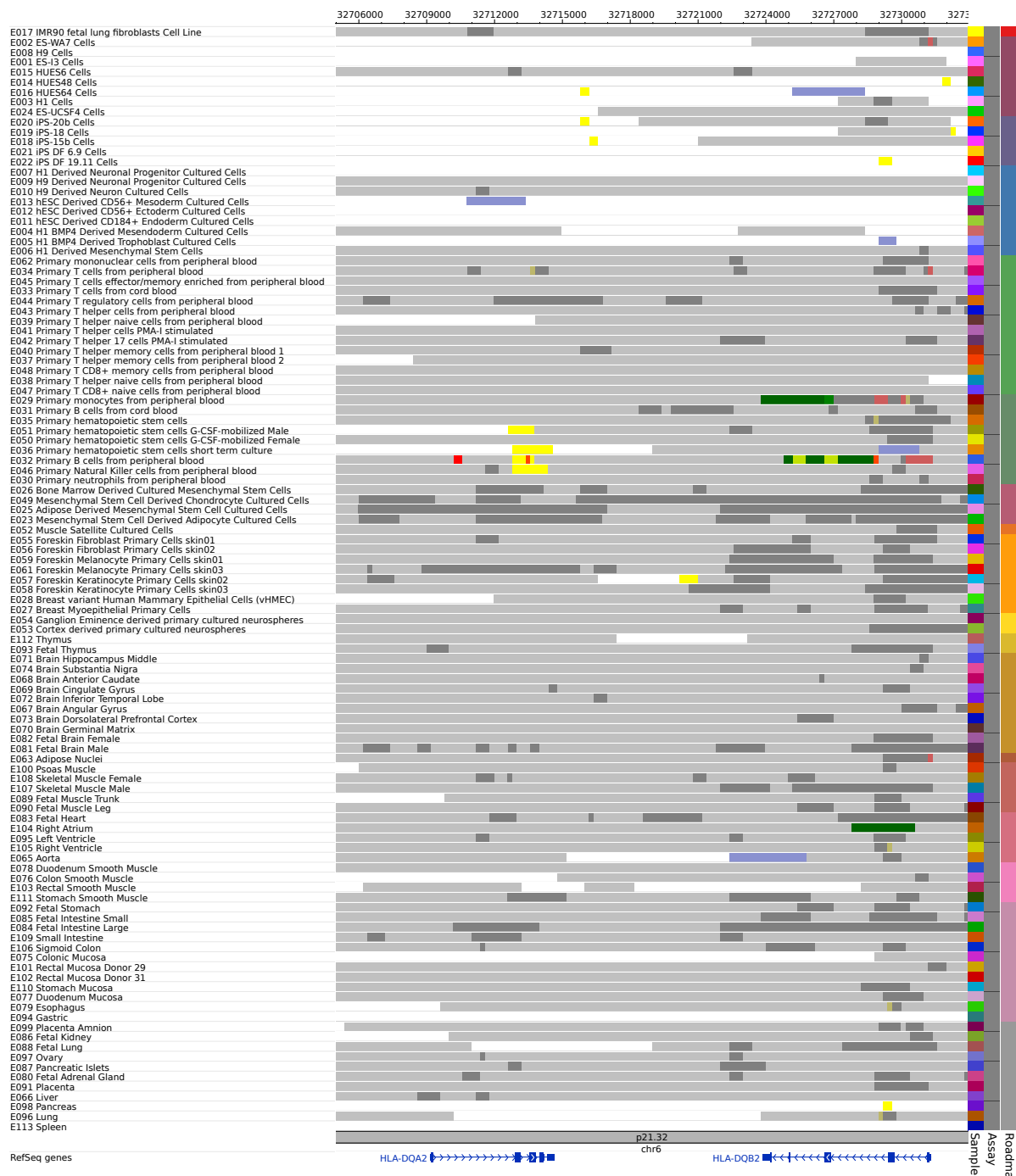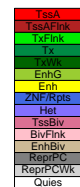

Supplement: S5 Fig — The map was generated using the Roadmap epigenome browser at http://epigenomegateway.wustl.edu/browser. (PDF) [file pone.0169918.s005.pdf]

**A**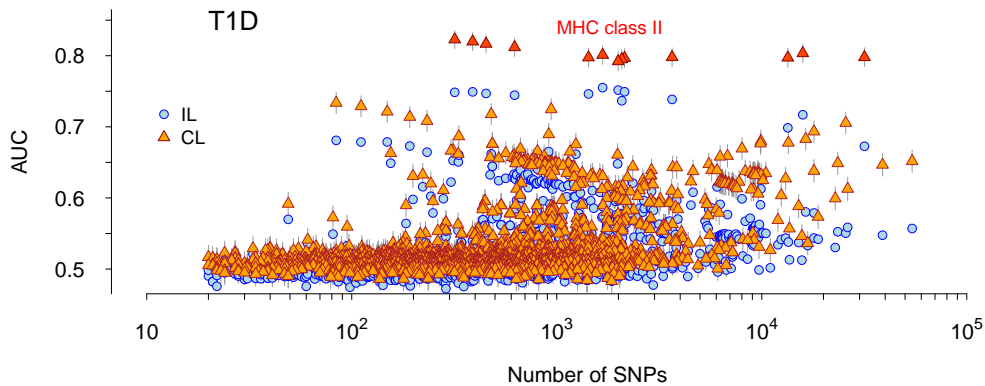**B**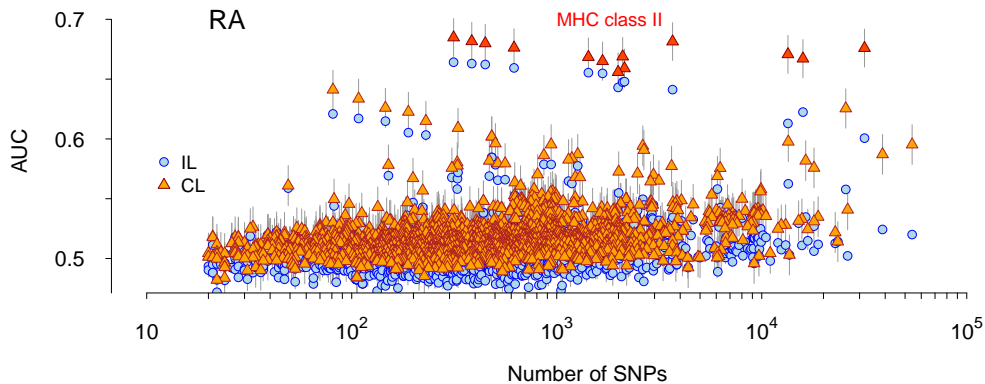

Supplement: S7 Fig — Inferences with and without interaction effects are shown together as functions of the number SNPs in each pathway. Pathways containing MHC class II genes are shown in red. Vertical lines are 95% c.i. IL, independent loci inference; CL, collective loci inference. (PDF) [file pone.0169918.s007.pdf]

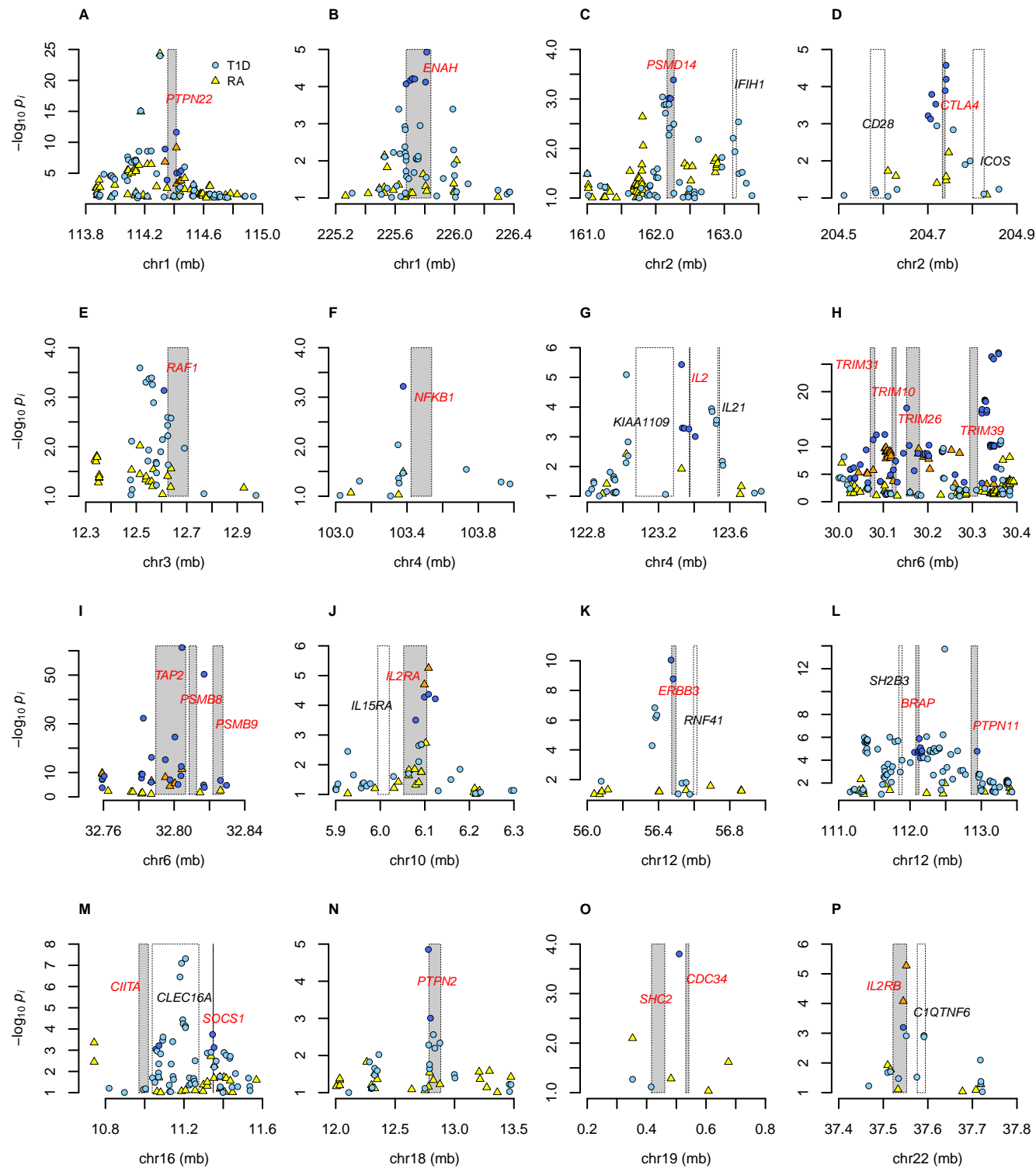

Supplement: S8 Fig — See Fig 4C. Genes shown in red are those in top-ranked pathways in Fig 7, whose coding regions are shown shaded in gray. Other genes of interest nearby are indicated with non-shaded coding regions. Darker blue (T1D) and orange (RA) symbols are the SNPs directly included in the pathways (within 50 kb of coding region and pi < 10−3). (PDF) [file pone.0169918.s008.pdf]

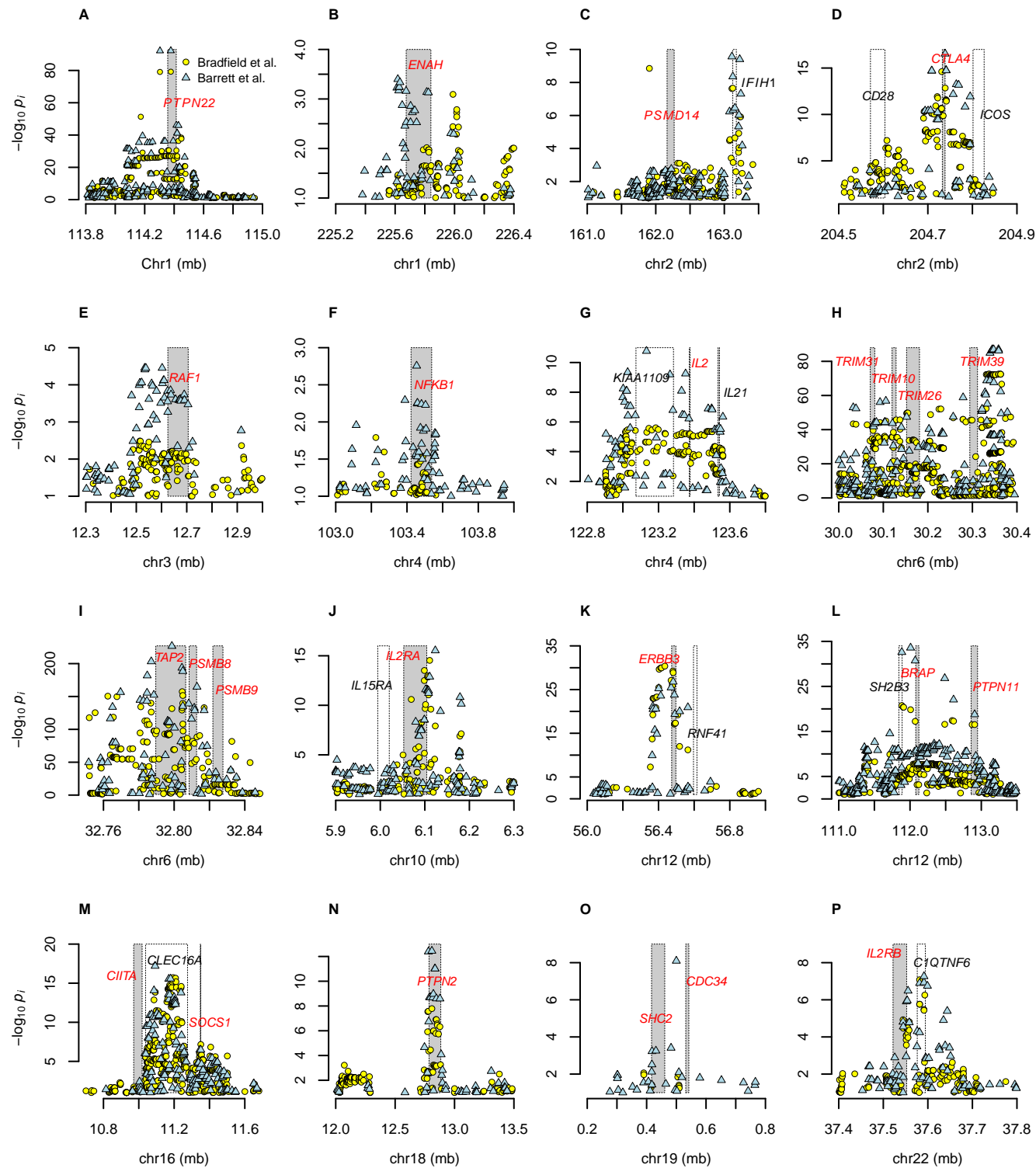

Supplement: S9 Fig — Data are from summary statistics of studies by Bradfield et al. [8] and Barrett et al. [10]. See S8 Fig for comparison. (PDF) [file pone.0169918.s009.pdf]
