# Supplementary material for: Collective Genetic Interaction Effects and the Role of Antigen-Presenting Cells in Autoimmune Diseases
Source: PLoS One. 2017 Jan 12;12(1):e0169918. doi: 10.1371/journal.pone.0169918 (PMC5231276; doi:10.1371/journal.pone.0169918)
Supplement: S6 Fig — (PDF) [file pone.0169918.s006.pdf]

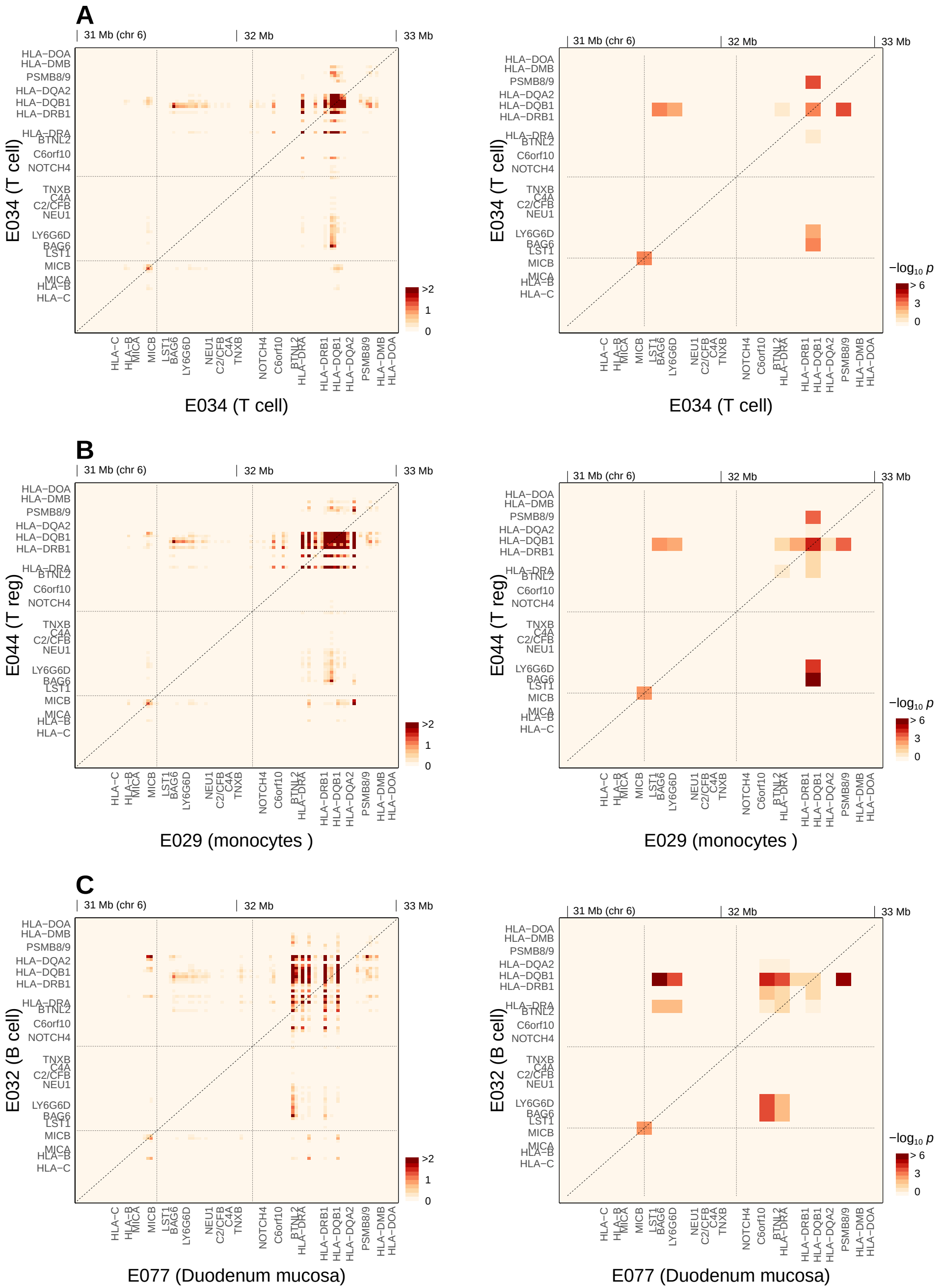

A
B
C
31 Mb (chr 6)
32 Mb
33 Mb
HLA−DOA
HLA−DMB
PSMB8/9
HLA−DQA2
HLA−DQB1
HLA−DRB1
HLA−DRA
BTNL2
C6orf10
NOTCH4
TNXB
C4A
C2/CFB
NEU1
LY6G6D
BAG6
LST1
MICB
MICA
HLA−B
HLA−C
E034 (T cell)
E044 (T reg)
E029 (monocytes)
E032 (B cell)
E077 (Duodenum mucosa)
>2
1
0
$-\log_{10} p$
> 6
3
0
